# Identification of a novel, expressed, alternatively spliced FCER1G protein that inhibits receptor function

**DOI:** 10.3389/fimmu.2026.1856092

**Published:** 2026-10-01

**Authors:** Andrew W. Gibson, Jianming Wu, R. Curtis Hendrickson, Travis Ptacek, Chaoling Dong, Charles E. Breeze, Jeffrey C. Edberg, Robert P. Kimberly

**Affiliations:** 1 Department of Medicine, The University of Alabama at Birmingham, Birmingham, AL, United States; 2 Department of Veterinary and Biomedical Sciences, University of Minnesota, Minneapolis, MN, United States; 3 Department of Microbiology, The University of Alabama at Birmingham, Birmingham, AL, United States; 4 Occupational and Environmental Epidemiology Branch, National Cancer Institute, Bethesda, MD, United States

**Keywords:** alternative splicing, Fc receptors (FcR), FCER1G, ITAM, myeloid cells

## Abstract

**Background:**

The ITAM-containing FcRγ chain, encoded by FCER1G, non-covalently couples with the immunoglobulin-binding receptors, -- FcγRI (CD64), FcγRIIIa (CD16), FcϵRI and FcαRI (CD89), -- to initiate intracellular signals and mediate phagocytosis, cytokine production and other functions. While functionally impactful variants have been identified in the ligand-binding alpha-chains of receptors associated with FcRγ, variants in the signal-generating FcRγ have not been explored. Signal modulation through structural FcRγ variants would have implications for FcRγ-associated receptors and their role in immune-mediated diseases.

**Methods:**

We carried out *in silico* analyses of whole genome DNA sequence databases based on short-read sequencing of ~76, 000 genomes, long-read sequencing of 1132 genomes, whole exome sequences of ~800, 000 participants and RNA transcript databases. We used the hg38 reference genome to identify genomic DNA and cDNA structural variants and Sanger sequencing of cDNAs derived from FcRγ mRNAs isolated from local participants to confirm ESTs in circulating immune cells. To detect a putative novel alternatively spliced translated protein (AS-FcRγ), we developed isoform-specific monoclonal antibodies to confirm AS-FcRγ co-immunoprecipitation with and support of receptor α-chain expression in transfection systems and primary cells. We assessed the function of the AS-FcRγ isoform by stimulus-induced cytokine production and degranulation.

**Results:**

In the human genome, insertions, deletions and rearrangements of FCER1G are absent, and with the exception of very rare single nucleotide variants, the FcRγ protein is monomorphic. Surprisingly, we confirmed that an alternatively spliced (AS) form of FcRγ message encodes a translated protein (AS-FcRγ) expressed in human myeloid cells. AS-FcRγ pairs with ligand binding FcR α-chains. The alternate exon 5’ in AS-FcRγ creates a novel cytoplasmic domain with a disrupted ITAM motif. Co-immunoprecipitation showed that AS-FcRγ associates with FcαRI in circulating neutrophils and mononuclear cells. A chimeric molecule consisting of the extracellular domain of FcαRI fused to the cytoplasmic domain of AS-FcRγ fails to stimulate cellular degranulation and cytokine production.

**Conclusion:**

AS-FcRγ is an expressed protein that associates with partner alpha-chains but is not signaling competent. It may represent a novel contributory mechanism mitigating the ability of Fc receptors to stimulate cellular signaling in myeloid cells with important implications for host defense and antibody-mediated autoimmune diseases.

## Introduction

Genetic variation is characteristic of many molecular elements in the immune system. These variants may affect physiological function and provide a way to modulate the intensity of the host response to pathogens and other stimuli (1, 2). Such heritable variants include single nucleotide polymorphisms (SNPs) as well as larger structural insertions, deletions and copy number variants which can affect host defense and may also predispose to autoimmune disease (3, 4). More recently, investigations have revealed an increasing role for alternate splicing of gene transcripts and the corresponding role that these mRNAs can play in expanding the range of protein isoforms that can be expressed (5, 6). These splice variants can also impact physiological function.

Among immune system receptors for antibody, single nucleotide polymorphisms in the ligand-binding α-chain can alter affinity (4, 7–9), glycosylation (10–12) and mobility in the plane of the cell membrane (13, 14). SNPs in the cytoplasmic domain can influence signaling responses to stimulation in experimental systems and primary cells (15, 16). Copy number variants can alter signal strength and net cell function (17). For several antibody receptors, the tyrosine activation motif (ITAM)-containing FcRγ chain, encoded by *FCER1G*, couples non-covalently with the ligand binding α-chain and transmits critical signals for receptor activation. Although some earlier studies suggested that there might be consequential genetic variation in FcRγ (18, 19), a careful study of this essential signal-transducing element has been side-tracked by the lack of suggestive association signals in disease-oriented GWAS studies (1). Such studies, however, are potentially constrained by the clinical phenotypes and populations studied.

We have carried out *in silico* analysis of whole genome and whole exome DNA sequence databases to identify single nucleotide and larger structural variations in *FCER1G*. We have assessed phased high-fidelity long-read whole genome assemblies from multiple ancestral backgrounds. In the human genome, insertions, deletions and rearrangements of *FCER1G* are absent, and with the exception of very rare single nucleotide variants, the FcRγ protein is monomorphic. However, we have identified an alternatively spliced (AS) protein incorporating an alternative exon 5 (exon 5’) which is expressed predominantly in myeloid cells and platelets and has some parallels to CD3-eta which is the alternatively spliced CD3-zeta protein with the deletion the membrane-distal, serine/threonine rich ITAM. With an intact transmembrane region identical to the wild type canonical FcRγ (WT-FcRγ) isoform, this AS-FcRγ protein can couple non-covalently with classical binding partners including FcαRI (CD89), FcγRIIIa (CD16a), FcγRI (CD64) and FcϵRI. Given the absence of an intact ITAM, AS-FcRγ does not transduce intracellular signals and, in the context of either homo-dimeric AS-FcRγ or heterodimeric AS-FcRγ/WT-FcRγ, it may mitigate the intensity of receptor induced signaling.

## Methods

### Donors

Donors were recruited at the University of Alabama at Birmingham (UAB). All protocols used were approved by UAB Institutional Review Board and all donors provided written informed consent. Anti-coagulated peripheral blood was obtained from normal healthy volunteers and SLE patients at UAB.

### Cell lines, and leukocytes and platelet preparations

The U937, HL-60 and THP-1 human monocytic cell lines, the HEK-293 cell line, and the wild type RBL-2H3 rat basophilic leukemic cell line were procured from ATCC (Manassas, VA). The FcRγ-deficient RBL-2H3 variant, which was derived from the wild type cell line by mutagenesis and which lacks endogenously expressed FcRγ, was previously described (20, 21). To isolate primary cells, freshly obtained anticoagulated blood was diluted 1:1 in PBS (pH 7.4) and centrifuged through a discontinuous two-step Ficoll-Hypaque gradient in 50-ml conical tubes. Peripheral blood mononuclear cells (PBMC) and normal density neutrophils (NDG, PMN) were harvested from the upper and lower Ficoll-Hypaque interfaces, respectively, washed three times with PBS (pH 7.4), resuspended, counted and used for mRNA preparation and for cell lysate preparation. Low density granulocytes (LDG) were harvested from the PBMC layer (22) and LDG purity (CD10^+/-^CD15^hi^CD14^lo^) was assessed at 85-90%. Human platelets were prepared as previously described (23). CD14^+^ monocytes, CD19^+^ B cells, CD4^+^and CD8^+^ T cells and CD56^+^ NK cells were stained with fluorochrome-conjugated cell-type specific antibodies and sorted by fluorescence-activated cell sorting (FACS). Post-sorting analysis determined that the cell-type purity was ~90%.

### Reagents

All monoclonal antibodies (mAbs) used were murine in origin. Anti-FcαRI (CD89) mAbs A59 and A77, and the anti-WT-FcRγ chain mAb 7D3 were previously described (23). The anti-AS-FcRγ mAb 4B1 and the pan-FcRγ mAb C20 were produced by the UAB Epitope Recognition and Immunoreagent Core Facility. Anti-human FcαRI mAb MIP8a was purchased from Bio-Rad (Hercules, California, USA) and the anti-actin mAb was from ThermoFisher Scientific (Grand Island, NY, USA). The F(ab’)_2_ preparations of mAbs A59 and A77 were produced at Rockland Immunochemicals (Limerick, PA), and the purity and integrity of the preparations was confirmed using SDS gel staining and Western Blot analysis. The AminoLink kit (CNBr activated Sepharose 4B coupling gel and reagents) was purchased from ThermoFisher. Protein G-Agarose and the protease inhibitor cocktail were from MilliporeSigma (St. Louis, MO, USA). The IL-4 cytokine detection ELISA kit and substrates were from BD Biosciences (Franklin Lakes, NJ, USA).

### Nucleic acid isolation

Genomic DNA was isolated using the Puregene DNA isolation kit (Qiagen, Germantown, MD, USA) as previously described (23). Total RNA was isolated from leukocytes using TRIzol™ total RNA isolation reagent (ThermoFisher). 5μg of total RNA were used to synthesize cDNA with the SuperScript™ Preamplification System (ThermoFisher).

### RT-PCR and cDNA sequencing of the alternatively spliced variant of FcRγ chain

Full-length AS-FcRγ RT-PCR was carried out with the forward primer (5’-CCC AAG ATG ATT CCA GCA GT-3’; anneals between nt 39 and nt 58) and the reverse primer (5’-CGA GCA GAT CAC GAG GTC AA-3’; anneals between nt 436 and nt 455) to yield a 416 base pair cDNA fragment (GenBank Accession Number for AS-FcRγ transcript: BG548748; Transcript ID: ENST00000367992.7). PCR reactions were carried out on a GeneAmp PCR System 9700 (ThermoFisher) using 2 μl of cDNA, 200 nM of each primer, 200 μM of dNTPs, 1.5 mM of MgCl_2_, and 2.5 U of Taq DNA polymerase in a 50-μl reaction volume. The PCR included one cycle of 95°C for 5 min, 35 cycles of denaturing at 94°C for 30 s, annealing at 55°C for 45 s, and extension at 72°C for 30 s, with a final extension at 72°C for 7 min. PCR products were electrophoresed on 2% agarose gels and purified using the QIAquick Gel Extraction Kit (Qiagen). Sanger sequencing of all PCR products in both directions used the BigDye Terminator Cycle Sequencing Kit (Version 3.1) (ThermoFisher). Additionally, the forward primer (5’- ACT GAA GAT CCA AGT GCG AAA G-3’; anneals between nt 179 and nt 199) and the reverse primer (5’-CGA GCA GAT CAC GAG GTC AA-3’; anneals between nt 436 and nt 455) were used in a second sensitive RT-PCR assay to yield a smaller AS-FcRγ cDNA fragment (277 bps) also for the detection of AS-FcRγ transcript in leukocyte populations.

### Determination of the poly(A) signal sequence in FCER1G for the AS-FcRγ splice variant

The 3’ RACE System (ThermoFisher) was used according to the vendor instructions to determine the 3’end poly(A) signal of AS-FcRγ cDNA. Briefly, the upper primer (5’-TAC ACG ATG GAG TCT CCC TCT ATC-3’), which anneals to AS-FcRγ cDNA, and the AUAP (Abridged Universal Amplification Primer) from 3’ RACE kit were used to amplify AS-FcRγ cDNAs. The amplified cDNA fragments were cloned into the pCR™2.1 vector using the TA Cloning Kit (ThemoFisher). cDNA clones of WT-FcRγ and AS-FcRγ were sequenced using BigDye Terminator Cycle Sequencing Kit.

### Generation of WT-FcRγ and AS-FcRγ expression constructs

All restriction enzymes were from Thermofisher. The human WT-FcRγ chain expression construct was generated by cloning BamHI/EcoRI-flanked 251-bps RT-PCR cDNA fragment containing the entire WT-FcRγ coding region into the eukaryotic expression vector pcDNA3.1 (ThermoFisher). The BamHI/EcoRI-flanked WT-FcRγ product was amplified from human mixed mononuclear cell cDNA. The upper primer 5’-CGC GGA TCC CAG CCC AAG ATG ATT CCA GCA GTG GTC -3’ (underlined nucleotides designate the BamHI restriction site) annealed at position 17 to 53 and the lower primer 5’-CCG GAA TTC CTA AAG CTA CTG TGG TGG TTT CTC ATG -3’ (underlined nucleotides designate the EcoRI restriction sites) annealed at position 266 to 286 (WT-FcRγ chain GenBank Accession Number: M33195; Transcript ID: ENST00000289902.2). Similarly, the human AS-FcRγ chain expression construct was generated by cloning BamHI/EcoRI-flanked 386-bps RT-PCR cDNA fragment containing the entire AS-FcRγ coding region into the eukaryotic expression vector pcDNA3.1. The BamHI/EcoRI-flanked AS-FcRγ RT-PCR product was amplified from human mixed mononuclear cell cDNA using the forward primer (5’-CGC GGA TCC CAG CCC AAG ATG ATT CCA GCA GTG GTC-3’; anneals between nt 36 and nt 62) and the reverse primer (5’- CCG GAA TTC CCA ACA TAG TGA AAC CCT GTC TC -3’; anneals between nt 403 and nt 422). All RT-PCR reactions for cloning were performed with KOD HiFi DNA Polymerase (MilliporeSigma). The sequences of all the cloned constructs were confirmed by direct sequencing in both directions.

### Generation of the FcαRI-EC-TM^R209L^-WT-FcRγ-CYT and FcαRI-EC-TM^R209L^-AS-FcRγ-CYT chimeric receptor expression constructs

The expression constructs for chimeric receptor comprised human FcαRI (CD89) extracellular (EC) domain and the transmembrane segment (TM) carrying the R209L mutation (FcαRI-EC-TM^R209L^) fused with the WT-FcRγ or AS-FcRγ cytoplasmic domain (CYT). The R209L mutation prevents the chimeric receptor from non-covalently coupling with FcRγ proteins in the plane of the membrane. Chimeric receptors were generated by overlapping PCR to create KpnI/EcoRI-flanked RT-PCR cDNA products. The resultant chimeric FcαRI-EC-TM^R209L^/WT-FcRγ-CYT and FcαRI-EC-TM^R209L^/AS-FcRγ-CYT cDNAs were cloned into pcDNA3.1. The FcαRI-EC-TM^R209L^ cDNA fragment was amplified from the FcαRI (CD89) pcDNA3.1 expression construct (15) with upper primer 5’-TAA CGG GGT ACC GGA GAC AAC ATG TGG TTC TTG ACA ACT-3’ (underlined nucleotides designate the KpnI restriction sites) and lower primer 5’-CTT TCG CAC TTG GAT CTT CAG ATT TTC AAC CAG TAT GGC CAA-3’. The WT-FcRγ-CYT cDNA fragment was amplified from the WT-FcRγ pcDNA3.1 expression construct using upper primer 5’-TTG GCC ATA CTG GTT GAA AAT CTG AAG ATC CAA GTG CGA AAG-3’ and lower primer 5’-CCG GAA TTC CTA AAG CTA CTG TGG TGG TTT CTC ATG-3’ (underlined nucleotides are EcoRI restriction site). The AS-FcRγ-CYT cDNA fragment was amplified with PCR from AS-FcRγ pcDNA3.1 expression construct using upper primer 5’-TTG GCC ATA CTG GTT GAA AAT CTG AAG ATC CAA GTG CGA AAG-3’ and (5’- CCG GAA TTC CCA ACA TAG TGA AAC CCT GTC TC-3’. The PCR fragments of FcαRI-EC-TM^R209L^ and FcRγ-CYTs were purified using the QIAquick Gel Extraction Kit and mixed with the upper primer (5’-TAA CGG GGT ACC GGA GAC AAC ATG TGG TTC TTG ACA ACT-3’) and lower primer 5’-CCG GAA TTC CTA AAG CTA CTG TGG TGG TTT CTC ATG -3’ to generate the FcαRI-EC-TM^R209L^-WT-FcRγ-CYT chimeric receptor. Similarly, purified PCR fragments of FcαRI-EC-TM^R209L^ and AS-FcRγ-CYT were mixed together with the upper primer (5’-TAA CGG GGT ACC GGA GAC AAC ATG TGG TTC TTG ACA ACT-3’) and lower primer (5’-CCG GAA TTC CCA ACA TAG TGA AAC CCT GTC TC-3’ to generate the FcαRI-EC-TM^R209L^-AS-FcRγ-CYT chimeric receptor. The RT-PCR reactions were performed using KOD HiFi DNA Polymerase. The purified PCR products were digested with KpnI and EcoRI restriction enzymes and subsequently cloned into the pcDNA3.1 vector. The sequences of all the constructed clones were confirmed by sequencing in both directions.

### Generation of the HA-WT-FcRγ and HA-AS-FcRγ expression constructs

The expression constructs for the N-terminal HA-tagged WT-FcRγ and AS-FcRγ were generated by cloning BglII/PstI-flanked RT-PCR products of WT-FcRγ and AS-FcRγ cDNAs into pDisplay vector (ThermoFisher) which encodes the HA tag. The WT-FcRγ cDNA fragment lacking the signal peptide sequence was amplified with upper primer 5’-CGCG AGA TCT CTG GGA GAG CCT CAG CTC TGC T-3’ (underlined nucleotides are BglII restriction site) and the lower primer 5’-AAA CTG CAG CTA AAG CTA CTG TGG TGG TTT CTC ATG-3’ (underlined nucleotides designate the PstI restriction sites). Similarly, the AS-FcRγ cDNA fragment lacking the signal peptide sequence was amplified using upper primer 5’-CGCG AGA TCT CTG GGA GAG CCT CAG CTC TGC T-3’ and the lower primer 5’-AAA CTG CAG CCA ACA TAG TGA AAC CCT GTC TC-3’. The purified PCR products were digested with BglII and PstI restriction enzymes and subsequently cloned into the pDisplay vector. The sequences of all the constructed clones were confirmed by sequencing from both directions.

### Generation of expression constructs for GST-FcRγ-CYT fusion protein

The GST-AS-FcRγ cytoplasmic domain fusion protein (GST-AS-FcRγ-CYT) expression constructs were generated by cloning BamHI/EcoRI-flanked RT-PCR products containing 144 bps of the unique AS-FcRγ cytoplasmic domain sequence into pGEX2T vector (GE Healthcare, Waukesha, WI, USA). The BamHI/EcoRI-flanked RT-PCR products were amplified using upper primer 5’-CAC GGA TCC TAC ACG ATG GAG TCT CCC TCT AT-3’ (underlined nucleotides are BamHI restriction sites) and lower primer 5’-CCG GAATTC CCA ACA TAG TGA AAC CCT GTC TC-3’ (underlined nucleotides are EcoRI restriction site). The RT-PCR reactions were performed using KOD HiFi DNA polymerase. The GST fusion constructs were transformed into E. coli DH5α and the fusion proteins were purified from bacterial lysates using glutathione sepharose beads (Cytiva, Marlsborough, MA, USA). The sequences of all the constructed clones including the plasmid junctions were confirmed by sequencing in both directions.

### Generation of RBL-2H3 cell lines stably expressing HA-WT-FcRγ, HA-AS-FcRγ, FcαRI-EC-TM^R209L^-WT-FcRγ-CYT and FcαRI-EC-TM^R209L^-AS-FcRγ-CYT chimeric receptors

The RBL-2H3 cells were maintained in the DMEM medium supplemented with 10% fetal calf serum (FBS) and 2 mM L-glutamine in 5% CO_2_. Individual transfections were carried out in 60 mm cell culture dishes at a cell density of 80-100% confluence. The FcαRI-EC-TM^R209L^-WT-FcRγ-CYT or FcαRI-EC-TM^R209L^-AS-FcRγ-CYT expression plasmid construct was purified using Wizard PureFection Plasmid DNA Purification System (Promega, Madison, WI, USA). 8 μg of plasmid DNA were used with 12 µl Lipofectamine 2000 reagent (GIBCO BRL) for transfection according to the manufacturer’s instructions. Transfected cells were cultured in DMEM medium supplemented with 10% fetal calf serum. G418 (1 mg/ml) was added to select stable transfectants, and G418 resistant cells were sorted by flow cytometry for comparable expression of all constructs.

### Degranulation assay

RBL-2H3 cells (10^5^ cells/well) expressing either FcαRI-EC-TM^R209L^-WT-FcRγ-CYT or FcαRI-EC-TM^R209L^-AS-FcRγ-CYT chimeric receptors were cultured overnight in 24-well culture plates (ThermoFisher). The culture media were removed from plates and the cells were incubated with DMEM medium containing 5 µg/ml F(ab’)_2_ fragments of anti-FcαRI mAb A59 for 45 minutes at 4 °C. The cells were washed with Tyrode’s buffer (130 mM NaCl, 5 mM KCl, 1.4 mM CaCl_2_, 1 mM MgCl2, 5.6 mM glucose, 10 mM HEPES, and 0.1% BSA, pH 7.4) and then stimulated with 20 μg/ml F(ab’)_2_ fragments of goat anti-mouse IgG in the Tyrode’s buffer. The supernatants were collected at 0, 15, 30, 45, and 60 minutes for measurement of β-hexosaminidase activity. Cells from control wells were lysed with a similar volume of Tyrode’s buffer containing 0.1% Triton X-100 to determine the total β-hexosaminidase activity for each RBL-2H3 stable cell line. Supernatants and cell lysates were incubated with substrate (1.3 mg/ml p-nitrophenyl-N-acetyl β-D-glucosamine) (MilliporeSigma) in 0.1 mM sodium citrate (pH 4.5) for 1 hour at 37 °C. The reaction was stopped by adding 0.2 mM sodium carbonate buffer (pH 10.0) and the enzyme reactivity was determined by measuring the optical density at 405 nm. The percentage of specific β-hexosaminidase activity released was calculated as follows: percentage release = 100 × average supernatant activity from 4 wells/average cell lysate activity from 4 control wells.

### Cytokine analysis

RBL-2H3 cells expressing either FcαRI-EC-TM^R209L^-WT-FcRγ-CYT or FcαRI-EC-TM^R209L^-AS-FcRγ-CYT were stimulated in 24-well tissue culture plates with surface-bound mAb A59 F(ab’)_2._ Wells were pre-coated with either anti-FcαRI mAb A59 F(ab’)_2_ (20 μg/ml) or with mIgG control F(ab’)_2_ (20 μg/ml) overnight at room temperature. The cells were added to the coated wells and the culture media were collected after 24 hours. For the FcRγ-deficient RBL-2H3 cells expressing HA-tagged WT-FcRγ or HA-AS-FcRγ, cells were opsonized in suspension with 40μg/ml anti-HA mAb 12CA5 or mIgE for 45 minutes on ice then stimulated by cross-linking with 20μg/ml goat anti-mouse IgG (H&L). Cells were then incubated for 24 hours in 24-well culture plates and cell culture supernatants were then collected. The concentration of secreted rat IL-4 was quantified by ELISA (BD Biosciences).

### Immunoprecipitation and western blotting detection of WT-FcRγ and AS-FcRγ

CD89 (FcαRI) was immunoprecipitated from cells using mAb A77 pre-bound to protein G-agarose, separated on PAGE gels and transferred to nitrocellulose membranes. FcRγ protein was blotted with anti-WT-FcRγ mAb 7D3 (21). AS-FcRγ protein (Uniprot accession number A6NCG8_Human) was detected with anti-AS-FcRγ mAb 4B1.

### Genomic analysis for single nucleotide variants

The population variant frequency variant call file (VCF) for chromosome 1 was downloaded for the Regeneron Million Exome cohort (24)(version 20231004; subset of 821, 979 unrelated donors from the 983, 578 donor cohort, which includes UK BioBank data), and variant impact was annotated with snpEff 5.0e using dbSNP 156 (25) and Ensembl (release 111) (26). Variants were filtered to non-synonymous coding or splicing variants in the *FCER1G* canonical transcript (ENST00000289902.2) with an allele frequency above 0.01%. Additionally, population variant frequency and Ensembl Variant Effect Predictor (27) impact results for the *FCER1G* locus (ENSG00000158869.11) was exported as from gnomAD v4.1.1 (28), which includes 76, 215 whole genomes and 730, 947 exomes (including UK BioBank data). The whole genome data was required because *FCER1G* exon5’ is not covered in exome sequencing. Variants were filtered to retain only those causing non-synonymous coding or splicing changes with an allele frequency above 0.05%.

### Genomic analysis of long read sequencing for larger structural variation

Publicly available samples were obtained from the Clinical Sequencing Evidence-Generating Research (CSER) consortium study, the Chinese pangenome project, the Arab pangenome project, NCBI’s Sequence Reads Archive (SRA), All of Us and combined with locally sequenced samples. In total, 1132 participants with either PacBio Circular Consensus Sequencing (CCS) reads or genomes assembled using PacBio CCS reads were used for analysis. The sources and any metadata available are found in supplementary data (**Supplementary Table 1**).

The pipelines for CSS assembly and scRNAseq were implemented in Snakemake (29). All pipelines were run using Snakemake 5.9.1, Anaconda 23.7.4 and python 3.6.6. The code for the pipelines and all of their associated scripts and configuration files can be found at https://github.com/KimberlyLab/2026-FCER1G-AS-Paper.

CCS libraries were *de-novo* assembled using hifiasm 0.20.0-r639 (30). The assemblies were annotated by alignment of sequence probes using BLAT (31). These probes included markers for the 5’ and 3’ coding sequences of *FCER1G* and for sequences upstream and downstream of *FCER1G*. Annotation reports generated by the pipeline were examined manually to determine whether there was evidence for *FCER1G* structural variants. Assemblies with possible larger structural variants were analyzed manually to verify the presence or absence of such variants. The sequences of the probes used are found in **Supplementary Table 2**.

### mRNA analysis: RT-qPCR

RT-qPCR was performed using 100 ng of cDNA in a 20 μL reaction and the standard 45 cycles in a 7900HT Fast Real-Time PCR System (ThermoFisher). Fluorescently labeled gene-specific primer-probe sets for WT-FcRγ, AS-FcRγ and the β-Actin internal control were from ThermoFisher. For WT-FcRγ: Forward primer, 5’-CAGCTATGAGA AATCAGATGGTGTTT-3’; Reverse primer, 5’-TCAGAGTCTCGTAAGTC TCCTGGTT-3’; Probe, 5’-FAM-CACGG GCCTGAGCA-MGBNFQ-3’. For AS-FcRγ: Forward primer, 5’-CAGC ATGAGAAATCAGATGGTGTTT-3’; Reverse primer, 5’-GCACTCCAGCCTGGTGA TAGA-3’; Probe, FAM-CACGATGGAGTCTCC-MGBNFQ-3’. The threshold cycles (Ct) for the amplifications were determined according to manufacturer’s recommendations. The Ct values were normalized relative to the internal actin control and the normalized values were used to calculate the fold difference using the 2^–ΔΔCt^ method.

### mRNA analysis: bulk cell data sets.

Bulk RNA-seq datasets were obtained for GEO GSE139360 (32) and dbGaP phs002063.v1.p1 (33). Reads were aligned to GRCh38 (primary assembly) with gencode.v31 using STAR 2.5.2a (34). We then compared the number of high confidence collapsed splice junction unique reads spanning the exon4 (ends at 161218723) to exon5 (starts at 161218723) or exon5-AS (starts at 161220389) and compared the ratio of exon5 to exon5-AS unique reads between samples.

### Single cell RNA-sequence analysis

Single cell RNA-seq (scRNA-seq) libraries were obtained from GEO datasets GSE188288 (35) and GSE269335 (36) and provided by Dr. Francis Lund (UAB). To detect alternatively spliced gamma chain transcripts (AS-FcRγ), we generated a modified gene annotation file that re-labeled the AS-FcRγ splice variant of *FCER1G* with a separate gene symbol, which enabled all downstream analysis programs to count AS-FcRγ and all other forms of FcRγ separately. Using this modified gene annotation, these datasets were reanalyzed using pipelines for Cell Ranger 9.0.1 (37) and Seurat 5.0.3 (38). The clustering data generated by Seurat was analyzed manually to assess the relative abundance of WT-FcRγ and AS-FcRγ in populations of monocytes and- neutrophils. Neutrophil subsets were defined as described by Wigerblad (35). Neutrophils were identified using *FCGR3B*, *CSFR3R* and *NAMPT* and were subsetted into NH0-NH3 subsets using *S100A12*, *S100A6*, *VIM*, *AIF1*, *TXNIP*, *CXCR2*, *MALAT1*, *NEAT1*, *CSF3R*, *IFIT1*, *HERC5* and *ISG15*. Monocyte subsets were defined as described by Dutertre (39). Monocytes were identified and subsetted using *CD14*, *CCL2*, *S100A9*, *FCGR3A*, *FCAR*, *VMO1*, *HLA*-*DRA* and *CD86*.

### Structural analyses of WT- and AS-FcRγ protein sequence

To identify putatively functional motifs in the unique sequence of AS-FcRγ, we used the Eukaryotic Linear Motif (ELM) (40) resource in addition to MotifFinders and Prosite search tools. Structural predictions for the canonical Fc receptor γ-chain (FcRγ) and its alternatively spliced isoform (AS-FcRγ) were generated using the AlphaFold 3 structural prediction server (Google DeepMind) (41). To characterize the structural alterations induced by the alternative splicing event, as well as potential interactions, full-length amino acid sequences for both isoforms were provided as input queries for structural modeling at https://alphafoldserver.com/. In addition, we modeled FcRγ Uniprot P30273) and AS-FcRγ (Uniprot A6NCQ8), as trimeric structures alongside the full length amino acid sequences of each of FcαRI (Uniprot 24071-1), FcγRIIIa (Uniprot P08637), FcγR1a (Uniprot P12314) and FcϵR1 (Uniprot P12319), (in WT-WT, AS-AS and WT-AS combinations).

## Results

The canonical *FcRγ is monomorphic and lacks large structural variants*.

The human FcRγ subunit is encoded by *FCER1G* (Gene ID: 2207) that contains five exons that span ~4 kilobases on chromosome 1q23.3 [NC_000001.11 (161, 215, 295.161, 219, 245) GRCh38.p14 Assembly]. The signal peptide (Sp) is encoded by exon 1 and part of exon 2. The remainder of exon 2 codes for the short extracellular domain (EC), the hydrophobic transmembrane region (TM), and the beginning of the cytoplasmic tail (CYT). The remainder of the CYT is encoded by exons 3, 4 and 5 (**Figure 1A**).

**Figure 1:**
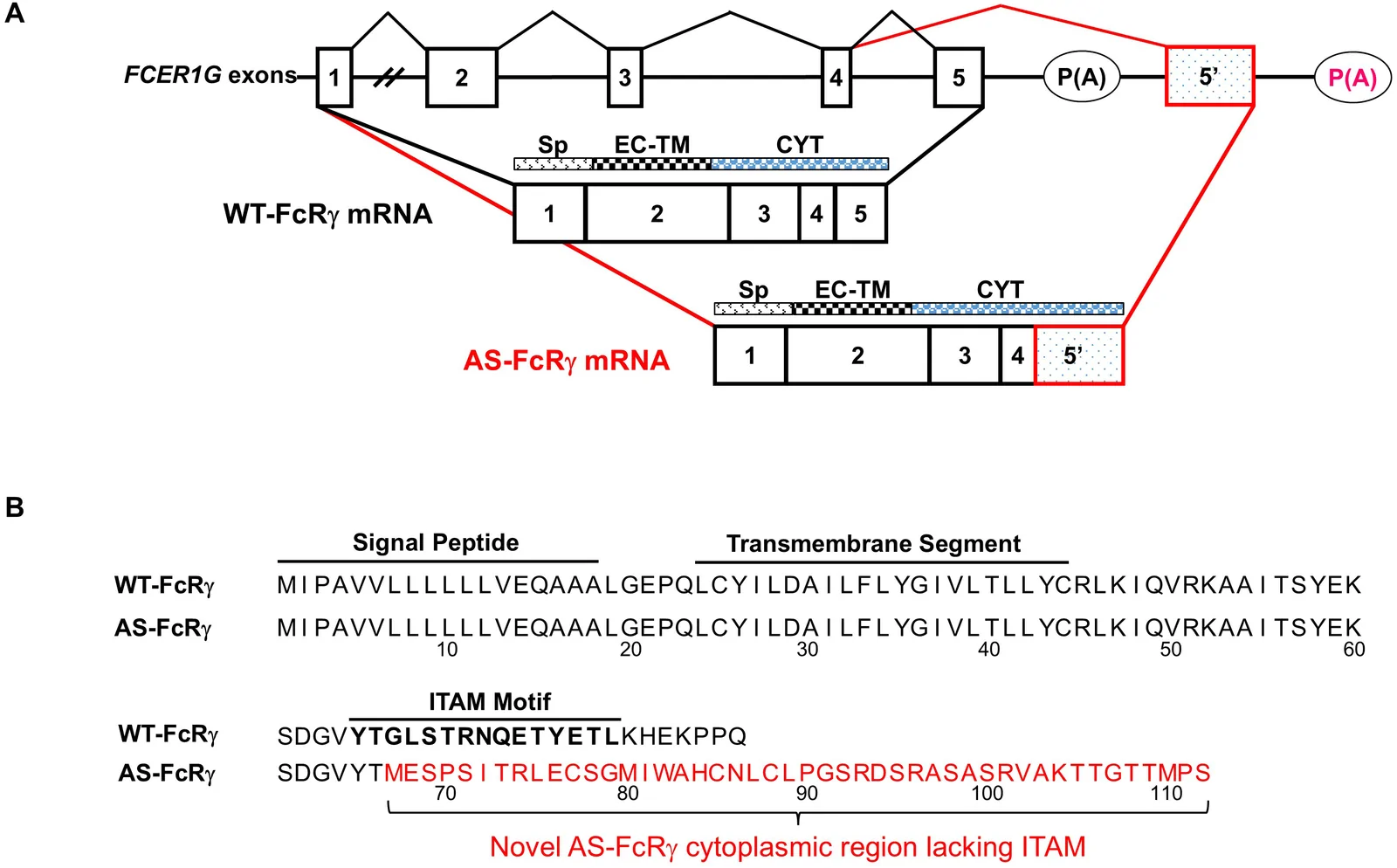
Structure of human *FCER1G* and gene products. **(A)** Genomic organization of *FCER1G* gene encoding canonical FcRγ (WT-FcRγ) and alternatively spliced FcRγ (AS-FcRγ). The signal peptide (Sp) is encoded by exon 1 and a part of exon 2. Exon 2 also encodes the extracellular and the transmembrane regions (EC-TM) in addition to the first four residues of the cytoplasmic tail (CYT). Exons 3, 4 and 5 primarily encode the WT-FcRγ CYT. *FCER1G* also codes for AS-FcRγ mRNA (GenBank # BG548748.1), however exon 5 is replaced by a downstream alternative exon 5 (exon 5’). **(B)** Protein sequence alignment of WT-FcRγ and AS-FcRγ. The WT-FcRγ cytoplasmic domain contains a canonical immunoreceptor tyrosine activation motif (ITAM). AS-FcRγ has a longer cytoplasmic domain compared to WT-FcRγ but does not contain an ITAM.

We performed *in silico* analyses of DNA sequence databases based on short read sequencing, including gnomAD with ~79, 000 whole genomes and both gnomAD and Regeneron with ~730, 000 and ~822, 000 whole exomes respectively both of which include UK BioBank samples. No non-synonymous coding single nucleotide polymorphisms with an allele frequency greater than 0.15% were identified in the canonical *FCER1G* transcript in the data sets studied. We identified one common SNP in exon 5’ of the alternately spliced transcript and several very rare non-synonymous SNPs (**Supplementary Figure 1**).

Recognizing that genomic copy number variation may influence quantitative biological function (42–46), we assessed 1132 whole genomes with long-read PacBio circular consensus sequencing and found no evidence for insertions, deletions or copy number variation.

### Identification of an alternatively spliced FcRγ (AS-FcRγ) variant mRNA in human leukocytes

Using the FcRγ mRNA reference sequence (GenBank # NM_004106.2), we carried out a BLAST search of the GenBank and dbEST databases. In addition to the canonical wild type FcRγ, we identified a cDNA sequence (GenBank # BG548748.1) containing a different terminal exon downstream of the *FCER1G* exon 4 and representing a putative novel protein splice form. We also identified a second FcRγ cDNA splice form lacking exon 2, which encodes the EC-TM, but we concluded that this splice form would not be predicted to generate a functional transmembrane protein since it lacks the TM sequence and would not be able to couple with ligand binding receptor alpha chains which is mediated by TM interactions. Therefore, we considered that the BG548748.1 transcript is likely the only expressed alternatively spliced FcRγ mRNA variant (AS-FcRγ) that might have functional consequence. As shown in **Figure 1A**, the WT-FcRγ and the AS-FcRγ mRNA transcripts are different in exon 5. The transcript encoding WT-FcRγ retains the traditional exon 5 that encodes the canonical ITAM signaling motif, but AS-FcRγ replaces that exon 5 with an alternative exon 5 (exon 5’) which lacks the ITAM (**Figure 1B**), suggesting that AS-FcRγ might be a non-signaling adaptor molecule. To verify the existence of AS-FcRγ mRNA, we carried out RT-PCR to amplify cDNAs of both WT-FcRγ and AS-FcRγ in peripheral blood mononuclear cells (PBMC), and polymorphonuclear leukocytes (PMN). In addition to WT-FcRγ, we detected variable levels of AS-FcRγ message in PBMC and PMN from most donors (**Figure 2**). AS-FcRγ message is also present in several myeloid cell lines (U937, HL-60 and THP-1) but is below detectable levels in the Jurkat T cells (**Supplementary Figure 2A**). We also found that AS-FcRγ mRNA was present in platelets (**Supplementary Figure 2B**). Using 3’-RACE analysis we determined the structure of the *FCER1G* mRNA coding for AS-FcRγ. The exon-intron junctions, exon/intron sizes and poly(A) signal of *FCER1G* coding for the canonical WT-FcRγ were determined from the GenBank Reference Sequence# NM_004106.2 (**Table 1A**). We sequenced 11 AS-FcRγ 3’-RACE clones and the results are summarized in **Table 1B**. Taken together, our data confirm that AS-FcRγ and canonical WT-FcRγ are different splice forms encoded by the same *FCER1G* gene.

**Figure 2:**
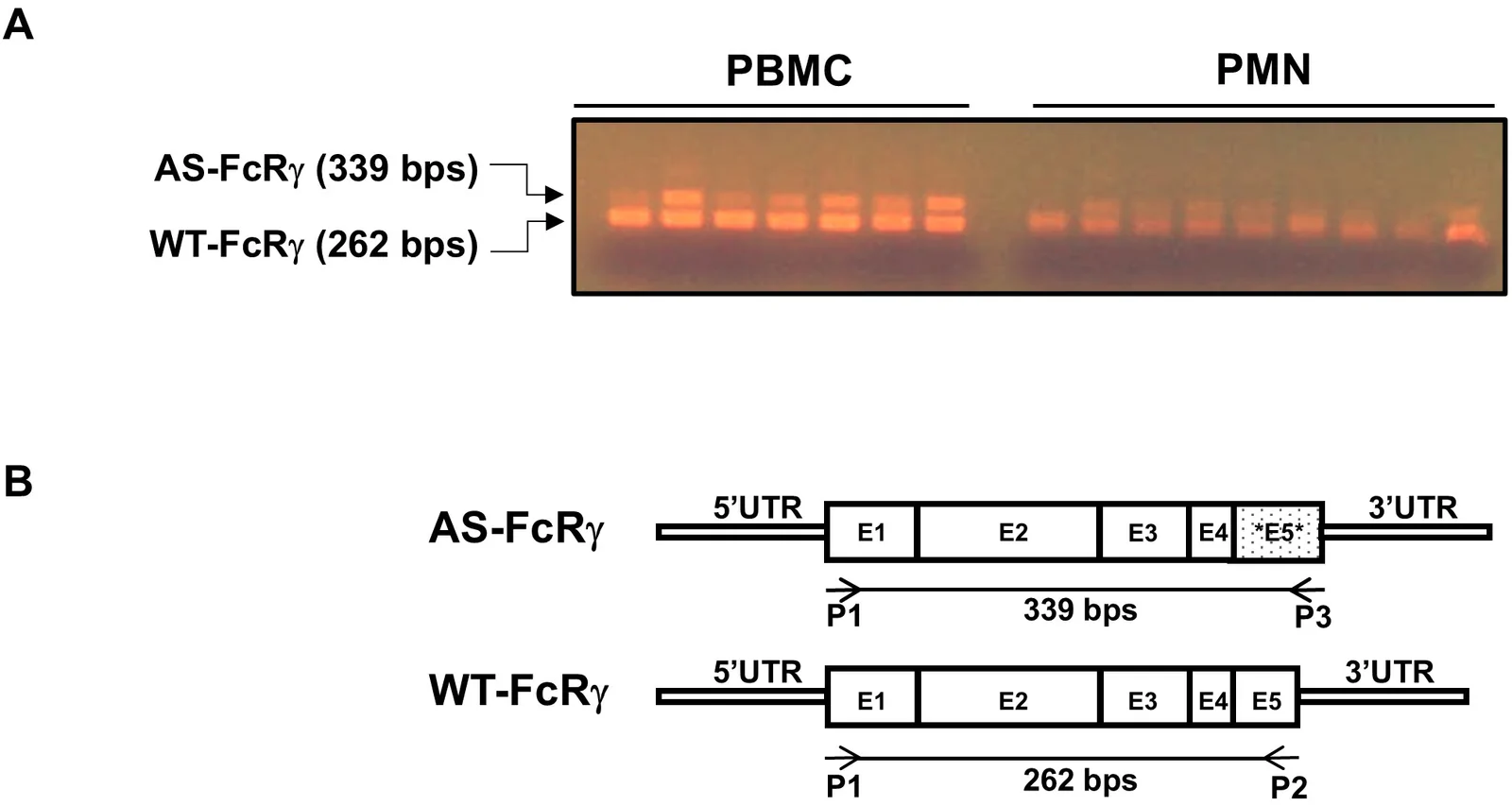
Detection of variable expression of WT-FcRγ and AS-FcRγ mRNA using RT-PCR. **(A)** Total RNA was extracted from cells and converted to cDNA. The cDNA was used to PCR-amplify FcRγ. RT-PCR products of WT-FcRγ (262 bps) and AS-FcRγ (339 bps) were detected in peripheral blood mononuclear cells (PBMC) and in polymorphonuclear leukocytes (PMN or neutrophils) collected from different donors. **(B)** Schematic locations of RT-PCR primers used in the detection analysis.

**Table 1:** Sequence of the Exon-Intron junctions of the human FCER1G gene.

a. Exon-intron junction of the human *FCER1G* coding for WT-FcRγ					
Exon			Intron		
Exon #	Size (bp)	5’ Splicing donor	Intron #	Size (bp)	3’ Splicing acceptor
1	49^a^	CAAGCAG gtaagag	1	2675	ccctcag CGGCCCT
2	92	ACTGAAG gtagcgc	2	163	attccag ATCCAAG
3	36	CTATGAG gtatgga	3	426	tctgcag AAATCAG
4	21	TTACACG gtaagtg	4	157	tctccag GGCCTGA
5	364	***AATAAA***ACTGCCATGGACTGG^b^			

b. Exon-intron junction of the human *FCER1G* gene coding for AS-FcRγ					
Exon			Intron		
Exon #	Size (bp)	5’ Splicing donor	Intron #	Size (bp)	3’ Splicing acceptor
1	49^a^	CAAGCAG gtaagag	1	2675	ccctcag CGGCCCT
2	92	ACTGAAG gtagcgc	2	163	attccag ATCCAAG
3	36	CTATGAG gtatgga	3	426	tctgcag AAATCAG
4	21	TTACACG gtaagtg	4	1666	ttttgag ATGGAGT
5	312	***AATAAA*** CTTTTATTGG^b^			

^a^ The size of exon 1 was counted from ATG start codon to the end of exon 1.

^b^ Guanidine is the last nucleotide to be transcribed at the mRNA 3’end that is followed by addition of poly-adenosines (GenBank Reference Sequence# NM_004106.2) or as determined by sequencing 11 RACE clones in AS-FcRγ 3’RACE analysis. The poly A signals are in bold.

### Detection of AS-FcRγ protein in human monocytes, neutrophils, and platelets

To determine whether AS-FcRγ message is expressed as a protein, we produced and validated a mouse monoclonal antibody (mAb 4B1) that recognizes the unique CYT region of AS-FcRγ (**Supplementary Figures 3A, B**). The 7D3 mAb which recognizes the canonical WT-FcRγ had been generated previously in our laboratory (21). In addition, we generated a mouse monoclonal antibody (mAb C20) that binds to a region common to both WT-FcRγ and AS-FcRγ (**Supplementary Figure 3C**). As shown in **Figure 3A**, both AS-FcRγ and WT-FcRγ were detected in CD14^+^ monocytes and in neutrophils (PMN). AS-FcRγ protein was also readily detected in human platelets (**Figure 3B**). Variable levels of AS-FcRγ protein were detected in low-density (L) and normal density (N) granulocytes (neutrophils) from SLE patients (**Supplementary Figure 4**). These data clearly demonstrate that the protein encoded by the alternatively spliced *FCER1G* mRNA (AS-FcRγ) is expressed in monocytes, neutrophils and platelets.

**Figure 3:**
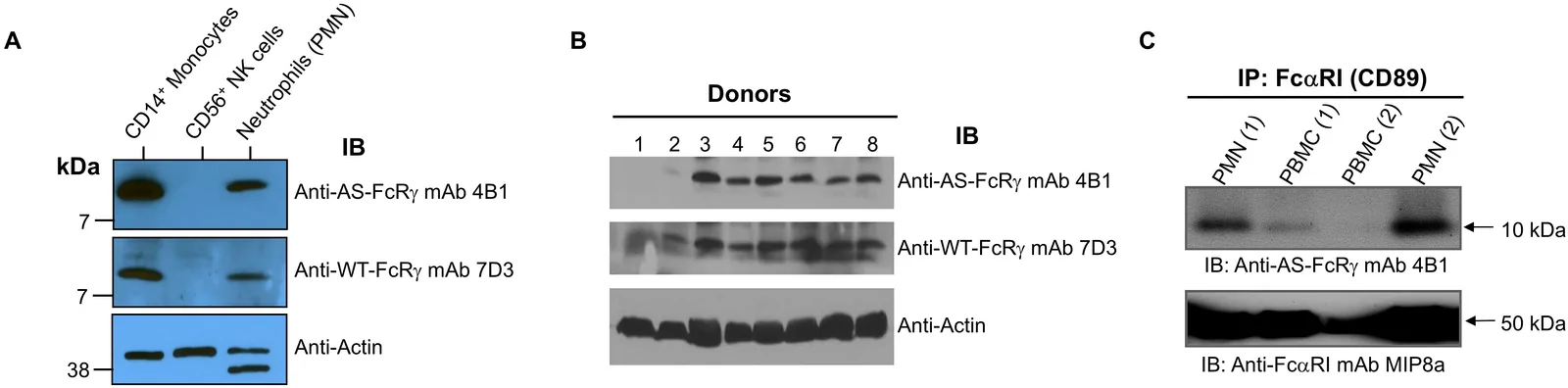
Western blot analysis of AS-FcRγ protein expression in immune cells. **(A)** Detection of AS-FcRγ and WT-FcRγ in subsets of human leukocytes. PBMCs were stained with a mixture of anti-CD3, anti-CD19, anti-CD14 and anti-CD56 fluorescently labeled antibodies and subsequently sorted by FACS to > 90% purity. Sorted cells were lysed, and 10 μg clarified lysate protein loaded onto SDS-PAGE gels and subjected to Western Blot analysis using anti-AS-FcRγ mAb 4B1 and anti-WT-FcRγ mAb 7D3 (for NK cell preparations only 2 μg lysate protein were loaded due to low cell number). Abundant AS-FcRγ and WT-FcRγ were detected in CD14^+^ monocytes and neutrophils from the same donor. AS-FcRγ protein in NK cells was below the level of detection in this assay. Data represent one of two experiments using different donors that gave similar results. **(B)** Variable levels of AS-FcRγ and WT-FcRγ proteins were detected in human platelets from several donors. **(C)** Co-precipitation of AS-FcRγ with FcαRI. FcαRI was immunoprecipitated from monocytes and neutrophils using anti-FcαRI mAb A77 and precipitates were immunoblotted with anti-AS-FcRγ mAb 4B1 and with anti-FcαRI mAb MIP8a (demonstrating overloading of FcαRI to maximize associated FcRγ detectability). Data represent one of 4 experiments giving similar results. Numbers (1) and (2) indicate the two donors from whom PBMC and neutrophils were isolated.

### AS-FcRγ associates with and supports the expression of Fcγ receptors.

The canonical WT-FcRγ adaptor protein non-covalently associates with several ligand-binding FcRs, including FcγRIIIa (CD16a), FcγRIa (CD64a) and FcεRIa, and is essential for their expression on the cell surface. Therefore, we wished to determine whether the AS-FcRγ adaptor protein could also couple with ligand-binding FcR alpha chains and stabilize their transmembrane expression on cells. We co-transfected HEK-293 cells with HA-AS-FcRγ or HA-WT-FcRγ together with *FCGR3A* or *FCGR1A* and stained co-transfected cells with anti-FcγRIIIa mAb 3G8 or anti-FcγRIa mAb 32.2, respectively. Cells were also stained with anti-HA mAb 12CA5 to show expression of HA-WT-FcRγ and HA-AS-FcRγ. Results show that, as with the canonical WT-FcRγ adaptor protein, the AS-FcRγ protein enhanced cell surface expression of FcγRIIIa (**Supplementary Figure 5A**) and FcγRIa (**Supplementary Figure 5B**). Additionally, both WT-FcRγ and AS-FcRγ increased the cell surface expression of rat FcϵRI when stably transfected into a mutant RBL-2H3 cell line shown to lack FcεRI surface expression which is dependent on the co-expression of FcRγ (20, 21) (**Supplementary Figure 6**). Taken together, these data show that the AS-FcRγ adaptor protein is able to associate with and support cell surface expression of FcγRIIIa, FcγRIa and FcεRIa. Several studies show that the WT-FcRγ adaptor protein also couples with the IgA-binding FcαRI receptor but is not required for surface expression of FcαRI on leukocytes (47–49). Recognizing that cell surface FcαRI might not be associated with either WT-FcRγ or AS-FcRγ, we wished, nonetheless, to determine whether endogenous AS-FcRγ could associate with FcαRI in primary human cells. As shown in **Figure 3C**, when FcαRI was immunoprecipitated from peripheral blood mononuclear cells (PBMC) and neutrophils (PMN), AS-FcRγ coimmunoprecipitated with FcαRI, suggesting a physical association between FcαRI and AS-FcRγ.

### AS-FcRγ is an activation-deficient accessory molecule

Since AS-FcRγ can associate with and support membrane expression of ligand-binding FcRs, we wished to determine whether AS-FcRγ could also signal for immune cell activation. We modeled both WT- and AS-FcRγ with corresponding FcR α-chains using AlphaFold3. The membrane-distal cytoplasmic domain of AS-FcRγ was disordered and we did not identify any predicted differences in the interactions of either WT- or AS-FcRγ with FcR α-chains (**Supplementary Figure 7**). Apart from the absence of the canonical ITAM in WT-FcRγ, bioinformatic analysis using the MotifFinder and Prosite search tools and the Eukaryotic Linear Motif (ELM) resource, indicated that the cytoplasmic domain of AS-FcRγ lacks known signaling motifs including a cryptic ITIM. To directly assess the functional capacity of AS-FcRγ, we used the FcRγ-deficient RBL-2H3 mutant cell line stably expressing either HA-WT-FcRγ or HA-AS-FcRγ. We opsonized cells with 40ug/ml anti-HA mAb, 12CA5, or mIgE (**Figure 4A**) and stimulated by cross-linking with goat-anti-mouse IgG (H&L). As shown in **Figure 4B** cells expressing WT-FcRγ signaled for significant production of IL-4 cytokine when stimulated with anti-HA or mIgE. However, when stimulated using the same experimental paradigm, cells expressing AS-FcRγ were unable to signal for IL-4 production. To confirm these data, we used the wild type FcRγ-sufficient RBL-2H3 cells to establish a second set of transfectant cell lines stably expressing FcαRI-WT-FcRγ and FcαRI-AS-FcRγ chimeric receptors consisting of the EC-TM of FcαRI fused to the CYT of WT-FcRγ or AS-FcRγ (**Figure 5A**). The chimeric receptors also carried the R209L amino acid mutation in the FcαRI TM, which prevents association with endogenous FcRγ proteins. Cells expressing comparable levels of FcαRI-WT-FcRγ and FcαRI-AS-FcRγ (**Figure 5B**) were opsonized with anti-FcαRI mAb A59 and stimulated by cross-linking with goat anti-mouse IgG (H&L). While stimulation of FcαRI-WT-FcRγ induced significant levels of degranulation of β-hexosaminidase, stimulation of FcαRI-AS-FcRγ failed to induce degranulation (**Figure 5C**). Cross-linking FcαRI-WT-FcRγ also induced robust IL-4 production while cross-linking of FcαRI-AS-FcRγ failed to induce IL-4 production (**Figure 5D**). Taken together, these data indicate that AS-FcRγ is a signaling deficient protein. These data also suggest that coupling of ligand-binding receptors with AS-FcRγ on the cell membrane could result in dampening receptor signaling.

**Figure 4:**
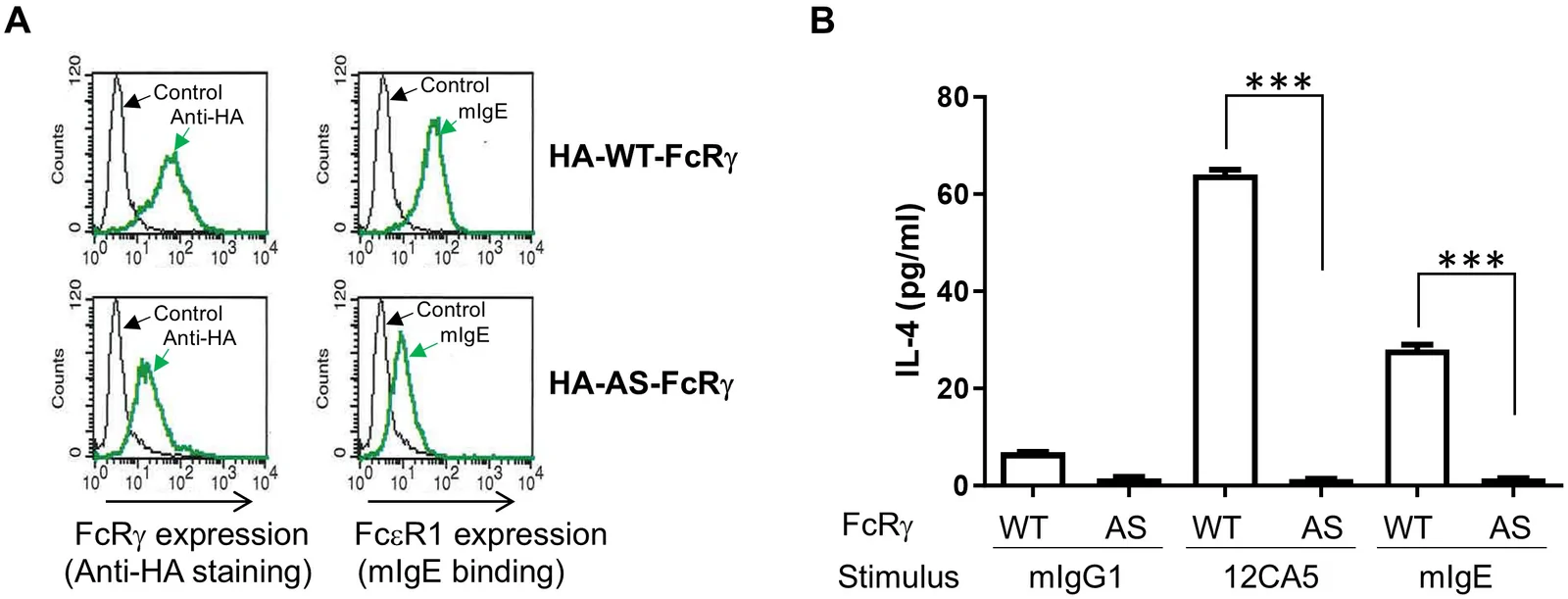
Analysis of AS-FcRγ function in FcRγ-deficient RBL-2H3 cells. **(A)** Stable expression of HA-tagged WT-FcRγ and HA-AS-FcRγ in FcRγ-deficient RBL-2H3 cells stained with anti-HA mAb 12CA5. Expression of FcεRIa which is dependent on the co-expression of FcRγ was also shown using mIgE. **(B)** HA-WT-FcRγ and HA-AS-FcRγ expressing cells were opsonized with 40μg/ml anti-HA mAb 12CA5 or mIgE and stimulated by cross-linking with 20μg/ml goat anti-mouse IgG. Cell culture media were collected after 24 hours and the concentration of secreted IL-4 was determined by ELISA. Stimulation of HA-WT-FcRγ expressing cells resulted in robust secretion of IL-4 compared with HA-AS-FcRγ expressing cells, which failed to induce secretion of IL-4 (***P < 0.001, Student’s t-test). Data represent one of three experiments giving similar results.

**Figure 5:**
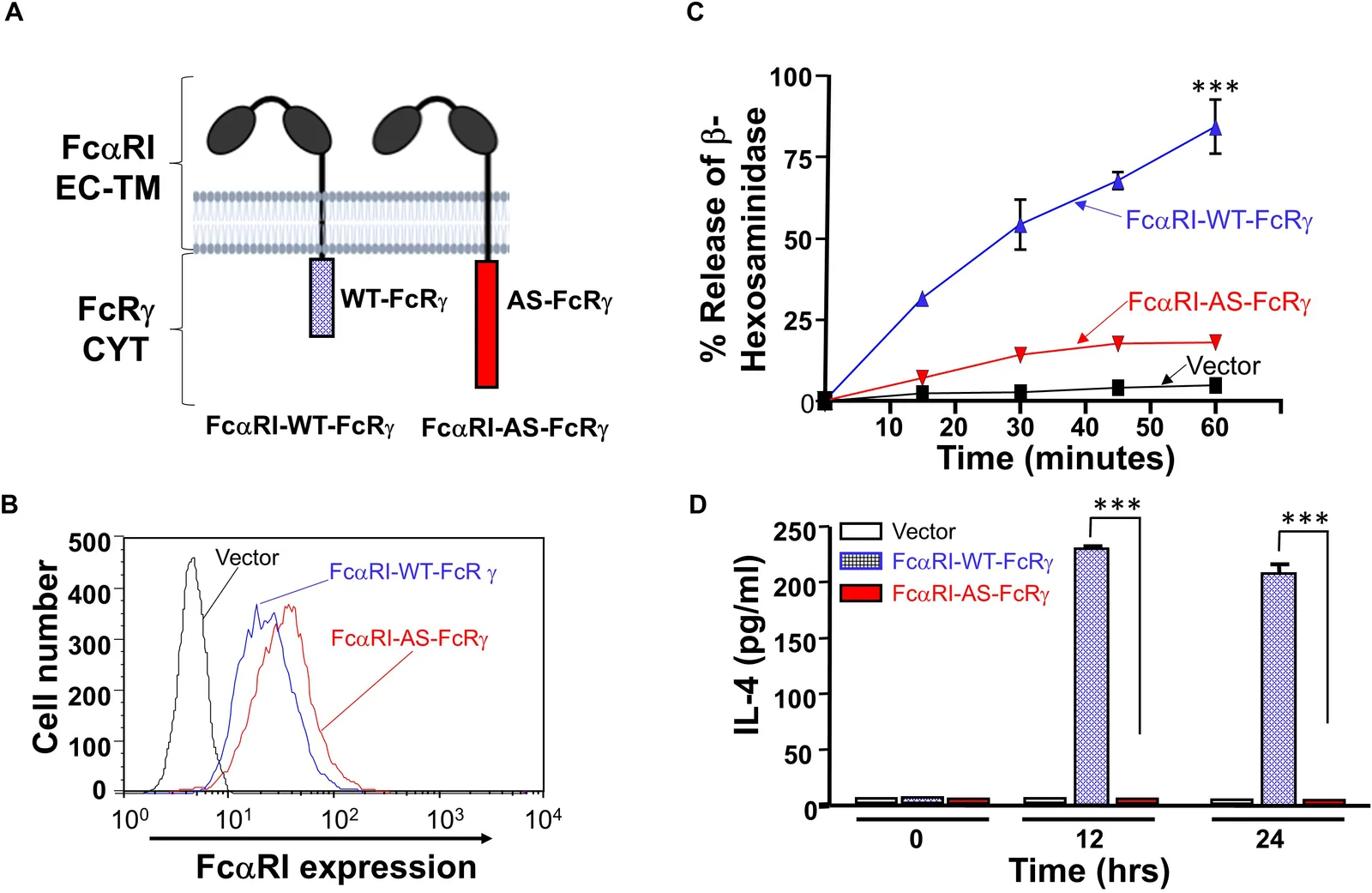
Analysis of AS-FcRγ function in FcRγ-sufficient RBL-2H3 cells. **(A)** Diagrams of the FcαRI-WT-FcRγ and FcαRI-AS-FcRγ chimeric receptors used (each with TM^209L^ as described in Materials and Methods). **(B)** Comparable surface expression of FcαRI-WT-FcRγ and FcαRI-AS-FcRγ in RBL-2H3. RBL-2H3 cells stably expressing the chimeric constructs were stained with anti-FcαRI mAb MIP8a and analyzed by FACS. **(C)** Stimulation of FcαRI-WT-FcRγ expressing cells in plates precoated with 40ug/ml mAb A59 induced robust degranulation of β-hexosaminidase and IL-4 production **(D)**, while similar stimulation of FcαRI-AS-FcRγ failed to induce degranulation or production of IL-4 cytokine (***p <0.001, Student’s t-test). These data represent one of 3 experiments giving similar results.

### Cell type and cell subset specific regulation of AS-FcRγ expression

Given the apparent variation in AS-FcRγ message and protein expression, we sought further data on cell-type specific expression of AS-FcRγ message. Using RT-qPCR and based on threshold cycle (Ct) values, we determined that WT-FcRγ mRNA expression was approximately 8-fold lower than the internal actin control (median 8-fold) in peripheral blood mononuclear cells (PBMCs). WT-FcRγ message was always more abundant than AS-FcRγ message but the ratios of the two transcripts varied across donors. In PBMCs the median ratio of WT- to AS-FcRγ message was ~900:1. In neutrophils the median ratio was ~300:1 (**Supplementary Table 3A**).

Recognizing that peripheral blood mononuclear cells are a heterogeneous mix of different cell populations with variable expression of both WT-FcRγ and AS-FcRγ (**Figure 3**), we used several large monocyte and neutrophil RNA-Seq data sets to explore WT- and AS-FcRγ message levels (32, 33, 35, 36). We confirmed that our analysis pipeline for RNA-Seq data was able to detect the presence of both the alternately spliced and canonical messages in both purified monocyte and neutrophil cell preparations. FcRγ-related message levels (proportionate number of positive reads) were typically higher in mononuclear cells (~2-fold more on average in monocytes, p=0.002, Wilcoxon rank sum test), with ratios for WT: AS-FcRγ of 100:1 to ~1500:1 for monocytes and neutrophils after filtering donors with no AS-FcRγ transcripts in the neutrophil data set (**Supplementary Table 3B**). When we compared the ratios of WT- to AS-FcRγ in the monocyte and neutrophil data sets, variability across donors was evident (**Supplementary Table 3C**).

Because of likely cell subpopulation heterogeneity within bulk cell preparations and considering that the variable ratios across individuals might reflect differential representation of such subpopulations, we accessed available single cell RNA-seq data sets for both monocytes (36) and peripheral blood neutrophils (32, 35). Interestingly, we noted that, at a message level, *FCAR* (encoding FcαRI; CD89) mRNA positive cells were more common in the classical monocyte cluster while *FCGR3A* (encoding FcγRIIIa; CD16a) mRNA positive cells were more common in the nonclassical monocyte clusters (**Figure 6**). *FCAR* mRNA single positive cells were more likely to be *FCER1G* negative than *FCGR3A* mRNA positive cells (χ^2^ >100; p<10^-4^), consistent with the ability of FcαR to be expressed in the absence of non-covalent pairing with FcRγ (48), unlike FcγRIIIa. Whether analyzed by proportionate transcript reads (**Supplementary Table 4**) or by the proportion of cells with detectable AS-FcRγ (**Figure 6**), monocytes in the non-classical cluster had a significantly higher proportion of AS-FcRγ reads and cells with detectable AS-FcRγ than cells in the classical cluster (χ^2^ >100; p<10^-4^) suggestive of a shift in splice variant transcription. No marked differences in WT- and AS-FcRγ message were seen across the four transcriptionally-defined neutrophil clusters, NH0-NH3 (35), (**Supplementary Figure 8**) perhaps due to the low levels of FcRγ message limiting the opportunity to see patterns of difference.

**Figure 6:**
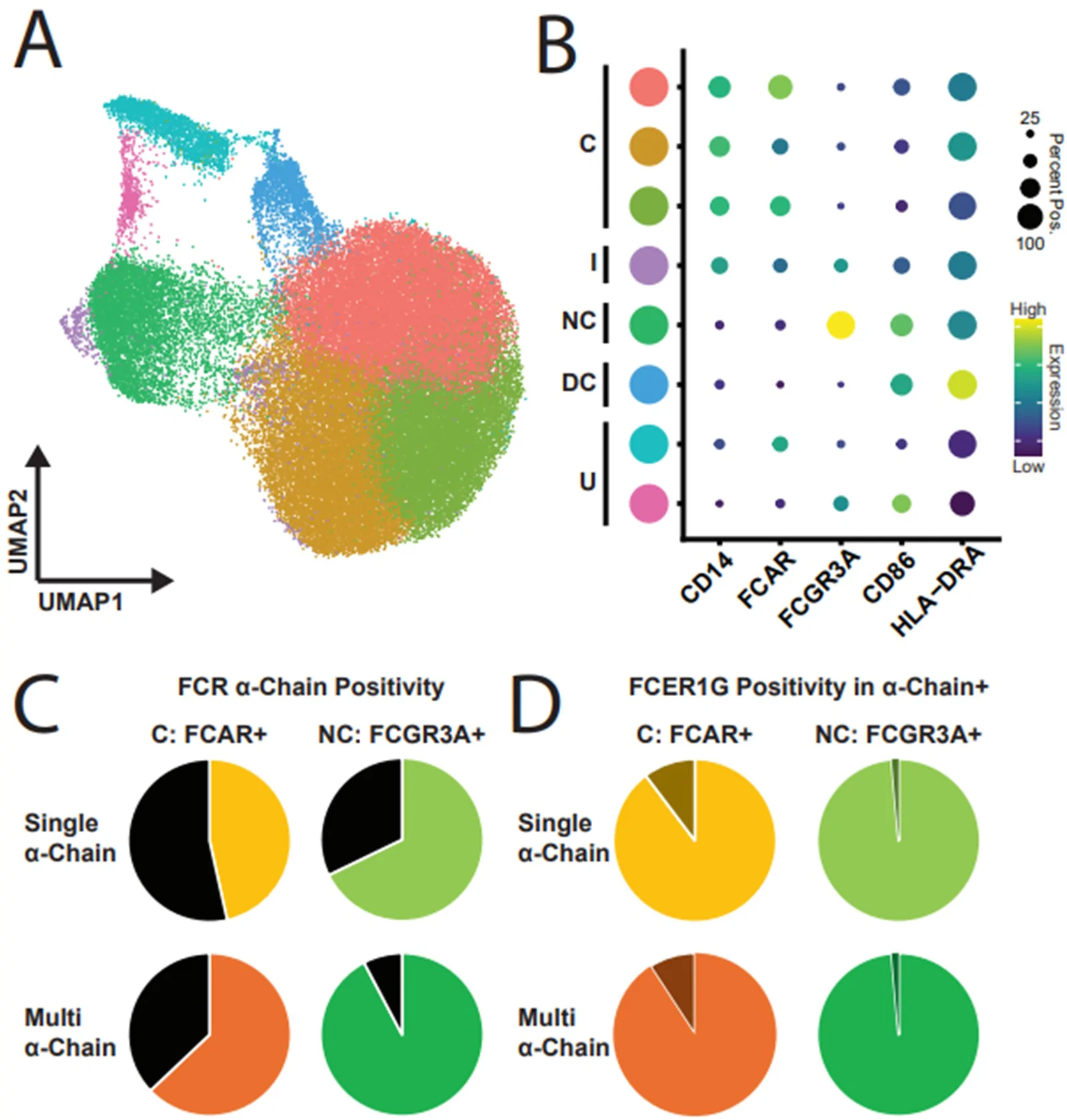
Single cell RNA-sequence analysis of FCER1G expression in monocytes. Single cell RNA-seq (scRNA-seq) libraries were obtained from GEO dataset GSE269335 (36) and from Dr. Francis Lund (UAB). As outlined in Methods, to detect alternatively spliced *FCER1G* chain transcripts (AS-FcRγ), a modified gene annotation file re-labeled the AS-FcRγ splice variant of *FCER1G* with a separate gene symbol, which enabled all downstream analysis programs to count AS-FcRγ and all other forms of FcRγ separately. **(A)** These datasets were reanalyzed using pipelines for Cell Ranger 9.0.1 (37) and Seurat 5.0.3 (38). **(B)** Monocyte subsets, as defined by Dutertre (39), were subsetted using *CD14*, *CCL2*, *S100A9*, *FCGR3A*, *FCAR*, *VMO1*, *HLA*-*DRA* and *CD86* expression; a simplified representation of these markers is illustrated in Panel B. Monocytes clusters are designated by C, classical; I, intermediate; NC, nonclassical; DC, dendritic cells; ~4% of cells were unclassified [U]). **(C)** Examining cells for *FCAR*, *FCGR3A* and *FCGR1A* (FcRγ-associated Fc receptors) transcripts, *FCAR* single positive cells were most prevalent (47%) in classical monocytes (Panel C, upper row; Single α-chain, shown in green). *FCAR* positivity with *FCGR3A* and/or *FCGR1A* positivity represented an additional 17% of classical cells (Panel C, lower row; Multi α-chain). *FCGR3A* single positive monocytes were most prevalent (68%) in nonclassical cells (shown in green). *FCGR3A* positive cells with *FCAR* and/or *FCGR1A* positivity were an additional 24%. **(D)** 10% of classical *FCAR* single positive monocytes were negative for both *FCER1G* transcripts whereas only 1% of nonclassical *FCGR3A* single positive monocytes were negative for both *FCER1G* transcripts. Strikingly, the proportion of the AS-FcRγ splice variant transcript positive cells compared to the WT-FcRγ positive cells was higher in the non-classical cluster (10.2%) than in the classical cluster (3.9%) (χ^2^ > 100; p < 10^-4^). These differences between classical and non-classical monocytes in *FCER1G* transcript-positive cells are replicated using total transcript reads (see **Supplementary Table 4**).

## Discussion

Genetic variation is characteristic of many molecular elements in the immune system. These variants may affect physiological function and provide a way to modulate the intensity of the host response to pathogens and other stimuli (1–4). Among immune system receptors for antibody, single nucleotide polymorphisms in the ligand-binding α-chain can alter affinity (3, 4, 7–9)glycosylation (10–12) and mobility in the plane of the cell membrane (13, 14). SNPs in the cytoplasmic domain can influence signaling responses to stimulation in experimental systems and primary cells (15, 16). Copy number variants can alter signal strength and net cell function (17, 50). For several antibody receptor molecular complexes, the tyrosine activation motif (ITAM)-containing FcRγ chain, encoded by *FCER1G*, couples non-covalently with the ligand binding α-chain in the plane of the cell membrane and transmits critical signals for receptor activation. Therefore, we have carried out *in silico* analysis of whole genome and whole exome DNA sequence databases to identify single nucleotide and larger structural variations in FcRγ. We have assessed phased high-fidelity long-read assemblies from multiple ancestral backgrounds. In the human genome, insertions, deletions and rearrangements of *FCER1G* are absent, and with the exception of very rare single nucleotide variants, the canonical FcRγ protein is monomorphic.

Recent literature has underscored the potential for alternative splicing events to substantially enhance proteomic diversity with impact on biological function (5, 6, 51–53). RNA sequencing of both bulk cell and single cell preparations has highlighted the multiplicity of messages, leading to some expressing skepticism about the role of alternative splicing in biology (54). However, a range of examples demonstrates both the expanding recognition of the role for proteins resulting from alternative splicing and also how these proteins may play a key role in the immune system (53). In examining the transcriptome for *FCER1G*, we noted a transcript (GenBank # BG548748.1) predicting an intact transmembrane domain but use of an alternative exon 5 (exon 5’ or exon 6 in some nomenclatures) which deleted the single ITAM in WT-FcRγ (**Figure 1**). This message was present in neutrophils, monocytes and peripheral blood mononuclear cells (**Figure 2**). Based on the unique predicted protein sequences of exon 5 and 5’, we generated monoclonal antibodies capable of detecting and distinguishing between both WT-FcRγ and AS-FcRγ isoforms (**Supplementary Figure 3**). AS-FcRγ protein was detected in myeloid cells (**Figure 3a**) although with some inter-individual variability (**Figure 3b**). Importantly, AS-FcRγ was associated with FcαRI (CD89), as demonstrated by co-immunoprecipitation (**Figure 3c**). These data support the notion that the translated protein product of the alternatively spliced message is positioned to influence the signaling function of FcαRI and perhaps other FcRγ-associated proteins, even in the presence of WT-FcRγ.

Without an intact ITAM and in the absence of a recognizable cryptic ITIM (55) or other recognized signaling motifs in AS-FcRγ, we hypothesized that the cytoplasmic domain of AS-FcRγ would not have the capacity to initiate intracellular signals leading to typical antibody-induced responses of myeloid cells, including granule release and cytokine production. Using extracellular N-terminal HA-tagged AS-FcRγ expressed in RBL-2H3 cells, we found no IL-4 production induced by cross-linking HA-tagged AS-FcRγ with the anti-HA mAb 12CA5. In contrast, HA-tagged WT-FcRγ transfectants showed robust IL4 production (**Figure 4**). Because the RBL-2H3 variant cells lack FcRγ intrinsically, this system also afforded the opportunity to assess if WT-FcRγ could reconstitute FcϵRI-initiated cytokine production. While both isoforms were able to support FcϵRI expression, only WT-FcRγ was able to support IL-4 production. The inability of AS-FcRγ to initiate cytokine production provides additional evidence that AS-FcRγ cannot initiate intracellular signaling events. We further explored AS-FcRγ function with chimeric constructs of FcαRI, replacing the cytoplasmic domain of FcαRI with either the cytoplasmic domain of WT-FcRγ or AS-FcRγ. With the WT-FcRγ chimera, we replicated the IL-4 production data generated with HA-tagged WT-FcRγ. Similarly, we confirmed our observations obtained with HA-tagged AS-FcRγ with the further demonstration of the lack of FcαRI-AS isoform chimera-initiated degranulation (**Figure 5**).

CD3-zeta (TCR ζ-chain; CD247), an FcRγ homologue, plays an integral role in TCR signaling. CD3-zeta has an alternatively spliced structural variant, CD3-eta, which deletes the membrane-distal third and final ITAM in the cytoplasmic domain, thereby reducing the number of intact ITAMs available for signal transduction (56, 57). Neither CD3-eta nor AS-FcRγ contain a cryptic ITIM (43, 55, 58), but unlike the retained ITAMs in CD3-eta, the deleted ITAM sequences of both CD3-eta and AS-FcRγ contain multiple serine and threonine residues in the 7-residue ITAM sequence between the membrane-proximal and membrane-distal YxxLs. We have previously shown that phosphorylation of several of these residues may play an active role in modulating the tyrosine-phosphorylation mediated signaling events initiated through these ITAMs in their respective host cells (21). In the TCR context with up to ten ITAMs contributed by CD3-δ, γ, ϵ and CD3-zeta (TCR ζ), only the ITAM deleted in CD3-eta has serine/threonines in the ITAM spacer region (**Supplementary Figure 9**). The complexity of this TCR signaling unit has focused current work on the contributions of the individual signaling molecules rather than single ITAM motifs. With this strategy, Love and colleagues have studied all of the TCR ζ ITAMs together rather than the single C-terminal ITAM which is deleted in the CD3-eta chain (59). The individual ITAMs seem to have differences in function (60) and a question for future work will be whether there might be a special functional role for the serine/threonine phosphorylation of the membrane-distal ITAM in CD3-zeta or wild-type FcRγ.

CD3-zeta message and protein expression vary with the context of cell stimulation including chronic autoimmune diseases (61–65), just as FcRγ expression varies with cytokine stimulation and cell activation (66). It is notable that AS-FcRγ message and protein also appear to vary across donors (**Figures 2**, **3**). Analysis of myeloid cell transcriptomes by both bulk and single cell RNA-Seq is consistent with this variation, and a deeper understanding of the correlates in terms of cell types, state of maturation and stimulation will be important for future work. Our observations that the proportion of WT-FcRγ and AS-FcRγ transcripts varies as monocytes transition from classical to non-classical subsets is consistent with a splice variant switch but might also reflect technical issues in read-depth in single cell data sets. We recognize the implications of AS-FcRγ for a number of cell receptor systems (67). Because the WT-FcRγ isoform is the only contributor of an ITAM module to the FcαRI, FcγRIa and FcγRIIIa receptor complexes, as examples, the substitution of the non-signaling AS-FcRγ isoform would likely have a major impact for receptor function. For other FcRγ binding partners lacking additional signaling modules such as GP VI (68–70), one could anticipate expressed receptors which are signaling deficient. Accordingly, AS-FcRγ may represent a novel mechanism mitigating the ability of AS-FcRγ associated receptors to stimulate cellular signals important for host defense and immune system functions.

There are a number of limitations in our current understanding of the physiological role of AS-FcRγ. Model systems including cell lines and engineered constructs may not capture the full range of cell functions, and although RBL cells have been used in many studies of cell biology and ITAM function (71), they are limited in the range of cytokines that they produce. Similarly, while studies of transcriptomics can provide substantial insight into cell function, direct prediction of corresponding protein levels is limited (72, 73). The lack of a cryptic ITIM and the absence of a new functional site or predicted molecular configuration in AS-FcRγ support the view that the absence of an ITAM determines the biology of AS-FcRγ. While not consistent with an active inhibitory process, as seen with Sab and FcαRI (16), an ITIM (74) or an ITAMi (75), the absence of an ITAM presumably mitigates a more robust, active recruitment and activation of Syk in receptor clusters. The stoichiometry of WT- and AS-FcRγ, whether as homo-dimers or hetero-dimers, in the receptor cluster may depend on the activation state of the cell and remains for future work in order to understand the full impact of this alternatively spliced protein isoform.

## Data Availability

The datasets presented in this study can be found in online repositories. The names of the repository/repositories and accession number(s) can be found in the article/**Supplementary Material**.

## References

[1] Ghodke-Puranik Y Olferiev M Crow MK . Systemic lupus erythematosus genetics: insights into pathogenesis and implications for therapy. Nat Rev Rheumatol. (2024) 20:635–48. doi: 10.1038/s41584-024-01152-2 39232240

[2] Metcalf CJE Graham AL Yates AJ Cummings DAT . Convergence and divergence of individual immune responses over the life course. Science. (2025) 389:604–9. doi: 10.1126/science.ady9543 40773563

[3] Li X Gibson AW Kimberly RP . Human FcR polymorphism and disease. Curr Top Microbiol Immunol. (2014) 382:275–302. doi: 10.1007/978-3-319-07911-0_13 25116105 PMC4209745

[4] Nagelkerke SQ Schmidt DE de Haas M Kuijpers TW . Genetic variation in low-to-medium-affinity Fcgamma receptors: functional consequences, disease associations, and opportunities for personalized medicine. Front Immunol. (2019) 10:2237. doi: 10.3389/fimmu.2019.02237 31632391 PMC6786274

[5] Park E Pan Z Zhang Z Lin L Xing Y . The expanding landscape of alternative splicing variation in human populations. Am J Hum Genet. (2018) 102:11–26. doi: 10.1016/j.ajhg.2017.11.002 29304370 PMC5777382

[6] Ule J Blencowe BJ . Alternative splicing regulatory networks: functions, mechanisms, and evolution. Mol Cell. (2019) 76:329–45. doi: 10.1016/j.molcel.2019.09.017 31626751

[7] Koene HR Kleijer M Algra J Roos D von dem Borne AE de Haas M . Fc gammaRIIIa-158V/F polymorphism influences the binding of IgG by natural killer cell Fc gammaRIIIa, independently of the Fc gammaRIIIa-48L/R/H phenotype. Blood. (1997) 90:1109–14. doi: 10.1182/blood.v90.3.1109.1109_1109_1114 9242542

[8] Salmon JE Edberg JC Kimberly RP . Fc gamma receptor III on human neutrophils. Allelic variants have functionally distinct capacities. J Clin Invest. (1990) 85:1287–95. doi: 10.1172/jci114566 1690757 PMC296565

[9] Wu J Edberg JC Redecha PB Bansal V Guyre PM Coleman K . A novel polymorphism of FcgammaRIIIa (CD16) alters receptor function and predisposes to autoimmune disease. J Clin Invest. (1997) 100:1059–70. doi: 10.1172/jci119616 9276722 PMC508280

[10] Edberg JC Barinsky M Redecha PB Salmon JE Kimberly RP . Fc gamma RIII expressed on cultured monocytes is a N-glycosylated transmembrane protein distinct from Fc gamma RIII expressed on natural killer cells. J Immunol. (1990) 144:4729–34. doi: 10.4049/jimmunol.144.12.4729 2141043

[11] Van Coillie J Schulz MA Bentlage AEH de Haan N Ye Z Geerdes DM . Role of N-glycosylation in FcgammaRIIIa interaction with IgG. Front Immunol. (2022) 13:987151. doi: 10.3389/fimmu.2022.987151 36189205 PMC9524020

[12] Washburn N Meccariello R Duffner J Getchell K Holte K Prod'homme T . Characterization of endogenous human FcgammaRIII by mass spectrometry reveals site, allele and sequence specific glycosylation. Mol Cell Proteomics. (2019) 18:534–45. doi: 10.1021/i560138a003 PMC6398215 30559323

[13] Li X Wu J Carter RH Edberg JC Su K Cooper GS . A novel polymorphism in the Fcgamma receptor IIB (CD32B) transmembrane region alters receptor signaling. Arthritis Rheum. (2003) 48:3242–52. doi: 10.1002/art.11313 14613290

[14] Xu L Xia M Guo J Sun X Li H Xu C . Impairment on the lateral mobility induced by structural changes underlies the functional deficiency of the lupus-associated polymorphism FcgammaRIIB-T232. J Exp Med. (2016) 213:2707–27. doi: 10.1084/jem.20160528 27799621 PMC5110019

[15] Wu J Ji C Xie F Langefeld CD Qian K Gibson AW . FcalphaRI (CD89) alleles determine the proinflammatory potential of serum IgA. J Immunol. (2007) 178:3973–82. doi: 10.4049/jimmunol.178.6.3973 17339498

[16] Gibson AW Wu J Hendrickson RC Ptacek T Mobley J Edberg JC . A genetically determined molecular switch modulates the anti-inflammatory potential of human IgA. Front Immunol. (2025) 16:1641351. doi: 10.3389/fimmu.2025.1641351 40936912 PMC12420274

[17] Breunis WB van Mirre E Geissler J Laddach N Wolbink G van der Schoot E . Copy number variation at the FCGR locus includes FCGR3A, FCGR2C and FCGR3B but not FCGR2A and FCGR2B. Hum Mutat. (2009) 30:E640–50. doi: 10.1002/humu.20997 19309690

[18] Feldner AC Turner AK Simpson JF Estus S . Skipping of FCER1G exon 2 is common in human brain but not associated with the Alzheimer's disease genetic risk factor rs2070902. J Alzheimers Dis Rep. (2023) 7:1313–22. doi: 10.3233/adr-230076 38143775 PMC10741965

[19] Wu J Edberg JC Gibson AW Kimberly RP . Conservation of FcepsilonRI gamma chain coding region in normals and in SLE patients. Lupus. (2002) 11:42–5. doi: 10.1191/0961203302lu145oa 11898918

[20] Bocek P Jr. Draberova L Draber P Pecht I . Characterization of Fc gamma receptors on rat mucosal mast cells using a mutant Fc epsilon RI-deficient rat basophilic leukemia line. Eur J Immunol. (1995) 25:2948–55. doi: 10.1002/eji.1830251035 7589096

[21] Shah S Gibson AW Ji C Darrington E Mobley J Kojima K . Regulation of FcRgamma function by site-specific serine phosphorylation. J Leukoc Biol. (2017) 101:421–8. doi: 10.1189/jlb.2ab0516-228r 27630214 PMC5235907

[22] Denny MF Yalavarthi S Zhao W Thacker SG Anderson M Sandy AR . A distinct subset of proinflammatory neutrophils isolated from patients with systemic lupus erythematosus induces vascular damage and synthesizes type I IFNs. J Immunol. (2010) 184:3284–97. doi: 10.4049/jimmunol.0902199 20164424 PMC2929645

[23] Qian K Xie F Gibson AW Edberg JC Kimberly RP Wu J . Functional expression of IgA receptor FcalphaRI on human platelets. J Leukoc Biol. (2008) 84:1492–500. doi: 10.1189/jlb.0508327 18784345 PMC2614599

[24] Sun KY Bai X Chen S Bao S Zhang C Kapoor M . A deep catalogue of protein-coding variation in 983,578 individuals. Nature. (2024) 631:583–92. doi: 10.1038/s41586-024-07556-0 38768635 PMC11254753

[25] Sherry ST Ward M Sirotkin K . dbSNP-database for single nucleotide polymorphisms and other classes of minor genetic variation. Genome Res. (1999) 9:677–9. doi: 10.1101/gr.9.8.677 10447503

[26] Harrison PW Amode MR Austine-Orimoloye O Azov AG Barba M Barnes I . Ensembl 2024. Nucleic Acids Res. (2024) 52:D891–9. doi: 10.1093/nar/gkad1049 37953337 PMC10767893

[27] McLaren W Gil L Hunt SE Riat HS Ritchie GR Thormann A . The ensembl variant effect predictor. Genome Biol. (2016) 17:122. doi: 10.1186/s13059-016-0974-4 27268795 PMC4893825

[28] Chen S Francioli LC Goodrich JK Collins RL Kanai M Wang Q . A genomic mutational constraint map using variation in 76,156 human genomes. Nature. (2024) 625:92–100. doi: 10.1038/s41586-023-06045-0 38057664 PMC11629659

[29] Koster J Rahmann S . Snakemake--a scalable bioinformatics workflow engine. Bioinformatics. (2012) 28:2520–2. doi: 10.1093/bioinformatics/bts480 22908215

[30] Cheng H Concepcion GT Feng X Zhang H Li H . Haplotype-resolved de novo assembly using phased assembly graphs with hifiasm. Nat Methods. (2021) 18:170–5. doi: 10.1038/s41592-020-01056-5 33526886 PMC7961889

[31] Kent WJ . BLAT--the BLAST-like alignment tool. Genome Res. (2002) 12:656–64. doi: 10.4324/9780203223673_chapter_8 11932250 PMC187518

[32] Mistry P Nakabo S O'Neil L Goel RR Jiang K Carmona-Rivera C . Transcriptomic, epigenetic, and functional analyses implicate neutrophil diversity in the pathogenesis of systemic lupus erythematosus. Proc Natl Acad Sci USA. (2019) 116:25222–33. doi: 10.1073/pnas.1908576116 31754025 PMC6911190

[33] Carlisle SM Qin H Hendrickson RC Muwanguzi JE Lefkowitz EJ Kennedy RE . Sex-based differences in the activation of peripheral blood monocytes in early Parkinson disease. NPJ Parkinsons Dis. (2021) 7:36. doi: 10.1038/s41531-021-00180-z 33850148 PMC8044127

[34] Dobin A Davis CA Schlesinger F Drenkow J Zaleski C Jha S . STAR: ultrafast universal RNA-seq aligner. Bioinformatics. (2013) 29:15–21. doi: 10.1093/bioinformatics/bts635 23104886 PMC3530905

[35] Wigerblad G Cao Q Brooks S Naz F Gadkari M Jiang K . Single-cell analysis reveals the range of transcriptional states of circulating human neutrophils. J Immunol. (2022) 209:772–82. doi: 10.4049/jimmunol.2200154 35858733 PMC9712146

[36] Bashore AC Xue C Kim E Yan H Zhu LY Pan H . Monocyte single-cell multimodal profiling in cardiovascular disease risk states. Circ Res. (2024) 135:685–700. doi: 10.1161/circresaha.124.324457 39105287 PMC11430373

[37] Zheng GX Terry JM Belgrader P Ryvkin P Bent ZW Wilson R . Massively parallel digital transcriptional profiling of single cells. Nat Commun. (2017) 8:14049. doi: 10.1038/ncomms14049 28091601 PMC5241818

[38] Hao Y Stuart T Kowalski MH Choudhary S Hoffman P Hartman A . Dictionary learning for integrative, multimodal and scalable single-cell analysis. Nat Biotechnol. (2024) 42:293–304. doi: 10.1038/s41587-023-01767-y 37231261 PMC10928517

[39] Dutertre CA Becht E Irac SE Khalilnezhad A Narang V Khalilnezhad S . Single-cell analysis of human mononuclear phagocytes reveals subset-defining markers and identifies circulating inflammatory dendritic cells. Immunity. (2019) 51:573–89:e8. doi: 10.1016/j.immuni.2019.08.008 31474513

[40] Kumar M Michael S Alvarado-Valverde J Zeke A Lazar T Glavina J . ELM-the Eukaryotic Linear Motif resource-2024 update. Nucleic Acids Res. (2024) 52:D442–55. doi: 10.1093/nar/gkad1058 37962385 PMC10767929

[41] Abramson J Adler J Dunger J Evans R Green T Pritzel A . Accurate structure prediction of biomolecular interactions with AlphaFold 3. Nature. (2024) 630:493–500. doi: 10.1038/s41586-024-07487-w 38718835 PMC11168924

[42] Conrad DF Pinto D Redon R Feuk L Gokcumen O Zhang Y . Origins and functional impact of copy number variation in the human genome. Nature. (2010) 464:704–12. doi: 10.1038/nature08516 19812545 PMC3330748

[43] Blank U Launay P Benhamou M Monteiro RC . Inhibitory ITAMs as novel regulators of immunity. Immunol Rev. (2009) 232:59–71. doi: 10.1111/j.1600-065x.2009.00832.x 19909356

[44] Frampton S Smith R Ferson L Gibson J Hollox EJ Cragg MS . Fc gamma receptors: their evolution, genomic architecture, genetic variation, and impact on human disease. Immunol Rev. (2024) 328:65–97. doi: 10.1111/imr.13401 39345014 PMC11659932

[45] Hollox EJ Zuccherato LW Tucci S . Genome structural variation in human evolution. Trends Genet. (2022) 38:45–58. doi: 10.1016/j.tig.2021.06.015 34284881

[46] Mao Y Harvey WT Porubsky D Munson KM Hoekzema K Lewis AP . Structurally divergent and recurrently mutated regions of primate genomes. Cell. (2024) 187:1547–60:e13. doi: 10.1016/j.cell.2024.01.052 38428424 PMC10947866

[47] Honorio-Franca AC Launay P Carneiro-Sampaio MM Monteiro RC . Colostral neutrophils express Fc alpha receptors (CD89) lacking gamma chain association and mediate noninflammatory properties of secretory IgA. J Leukoc Biol. (2001) 69:289–96. doi: 10.1189/jlb.69.2.289 11272280

[48] Launay P Patry C Lehuen A Pasquier B Blank U Monteiro RC . Alternative endocytic pathway for immunoglobulin A Fc receptors (CD89) depends on the lack of FcRgamma association and protects against degradation of bound ligand. J Biol Chem. (1999) 274:7216–25. doi: 10.1074/jbc.274.11.7216 10066783

[49] Maliszewski CR March CJ Schoenborn MA Gimpel S Shen L . Expression cloning of a human Fc receptor for IgA. J Exp Med. (1990) 172:1665–72. doi: 10.1084/jem.172.6.1665 2258698 PMC2188749

[50] Schmidt DE Heitink-Polle KMJ Laarhoven AG Bruin MCA Veldhuisen B Nagelkerke SQ . Transient and chronic childhood immune thrombocytopenia are distinctly affected by Fc-gamma receptor polymorphisms. Blood Adv. (2019) 3:2003–12. doi: 10.1182/bloodadvances.2019000068 31270082 PMC6616256

[51] Liu Q Fang L Wu C . Alternative splicing and isoforms: From mechanisms to diseases. Genes (Basel). (2022) 13. doi: 10.3390/genes13030401 35327956 PMC8951537

[52] Marasco LE Kornblihtt AR . The physiology of alternative splicing. Nat Rev Mol Cell Biol. (2023) 24:242–54. doi: 10.1038/s41580-022-00545-z 36229538

[53] Ueda MT Inamo J Miya F Shimada M Yamaguchi K Kochi Y . Functional and dynamic profiling of transcript isoforms reveals essential roles of alternative splicing in interferon response. Cell Genom. (2024) 4:100654. doi: 10.1016/j.xgen.2024.100654 39288763 PMC11602592

[54] Tress ML Abascal F Valencia A . Alternative splicing may not be the key to proteome complexity. Trends Biochem Sci. (2017) 42:98–110. doi: 10.1016/j.tibs.2016.08.008 27712956 PMC6526280

[55] Barrow AD Trowsdale J . You say ITAM and I say ITIM, let's call the whole thing off: the ambiguity of immunoreceptor signalling. Eur J Immunol. (2006) 36:1646–53. doi: 10.1002/eji.200636195 16783855

[56] Clayton LK D'Adamio L Howard FD Sieh M Hussey RE Koyasu S . CD3 eta and CD3 zeta are alternatively spliced products of a common genetic locus and are transcriptionally and/or post-transcriptionally regulated during T-cell development. Proc Natl Acad Sci USA. (1991) 88:5202–6. doi: 10.1073/pnas.88.12.5202 1828894 PMC51840

[57] Jin YJ Clayton LK Howard FD Koyasu S Sieh M Steinbrich R . Molecular cloning of the CD3 eta subunit identifies a CD3 zeta-related product in thymus-derived cells. Proc Natl Acad Sci USA. (1990) 87:3319–23. doi: 10.1073/pnas.87.9.3319 2139725 PMC53891

[58] Waterman PM Cambier JC . The conundrum of inhibitory signaling by ITAM-containing immunoreceptors: potential molecular mechanisms. FEBS Lett. (2010) 584:4878–82. doi: 10.1016/j.febslet.2010.09.029 20875413 PMC2998577

[59] Love PE Hatzihristidis T Bryant J Gaud G . Early events in TCR signaling - the evolving role of ITAMs. Front Immunol. (2025) 16:1563049. doi: 10.3389/fimmu.2025.1563049 40342420 PMC12058738

[60] Majumdar S Echelibe H Bettini M Bettini ML . The impact of CD3zeta ITAM multiplicity and sequence on CAR T-cell survival and function. Front Immunol. (2024) 15:1509980. doi: 10.3389/fimmu.2024.1509980 39885989 PMC11779709

[61] Ciszak L Pawlak E Kosmaczewska A Potoczek S Frydecka I . Alterations in the expression of signal-transducing CD3 zeta chain in T cells from patients with chronic inflammatory/autoimmune diseases. Arch Immunol Ther Exp (Warsz). (2007) 55:373–86. doi: 10.1007/s00005-007-0042-6 18060371

[62] Euren A Lynch K Lindfors K Parikh H Koletzko S Liu E . Risk of celiac disease autoimmunity is modified by interactions between CD247 and environmental exposures. Sci Rep. (2024) 14:25463. doi: 10.1038/s41598-024-75496-w PMC11567144 39462122

[63] Nambiar MP Enyedy EJ Fisher CU Warke VG Juang YT Tsokos GC . Dexamethasone modulates TCR zeta chain expression and antigen receptor-mediated early signaling events in human T lymphocytes. Cell Immunol. (2001) 208:62–71. doi: 10.1006/cimm.2001.1761 11277620

[64] Nambiar MP Juang YT Krishnan S Tsokos GC . Dissecting the molecular mechanisms of TCR zeta chain downregulation and T cell signaling abnormalities in human systemic lupus erythematosus. Int Rev Immunol. (2004) 23:245–63. doi: 10.1080/08830180490452602 15204087

[65] Takeuchi T Suzuki K Kondo T Yoshimoto K Tsuzaka K . CD3 zeta defects in systemic lupus erythematosus. Ann Rheum Dis. (2012) 71:i78–81. doi: 10.1136/annrheumdis-2011-200641 22460144

[66] Cameron AJ Harnett MM Allen JM . Differential recruitment of accessory molecules by FcgammaRI during monocyte differentiation. Eur J Immunol. (2001) 31:2718–25. doi: 10.1007/978-1-4842-1323-0_7 11536170

[67] Hamerman JA Ni M Killebrew JR Chu CL Lowell CA . The expanding roles of ITAM adapters FcRgamma and DAP12 in myeloid cells. Immunol Rev. (2009) 232:42–58. doi: 10.1111/j.1600-065x.2009.00841.x 19909355 PMC3248395

[68] Jandrot-Perrus M Busfield S Lagrue AH Xiong X Debili N Chickering T . Cloning, characterization, and functional studies of human and mouse glycoprotein VI: a platelet-specific collagen receptor from the immunoglobulin superfamily. Blood. (2000) 96:1798–807. doi: 10.1182/blood.v96.5.1798 10961879

[69] Nieswandt B Bergmeier W Schulte V Rackebrandt K Gessner JE Zirngibl H . Expression and function of the mouse collagen receptor glycoprotein VI is strictly dependent on its association with the FcRgamma chain. J Biol Chem. (2000) 275:23998–4002. doi: 10.1074/jbc.m003803200 10825177

[70] Tsuji M Ezumi Y Arai M Takayama H . A novel association of Fc receptor gamma-chain with glycoprotein VI and their co-expression as a collagen receptor in human platelets. J Biol Chem. (1997) 272:23528–31. doi: 10.1074/jbc.272.38.23528 9295288

[71] Paolini R Numerof R Kinet JP . Phosphorylation/dephosphorylation of high-affinity IgE receptors: a mechanism for coupling/uncoupling a large signaling complex. Proc Natl Acad Sci USA. (1992) 89:10733–7. doi: 10.1073/pnas.89.22.10733 1438270 PMC50416

[72] Liu Y Beyer A Aebersold R . On the dependency of cellular protein levels on mRNA abundance. Cell. (2016) 165:535–50. doi: 10.1016/j.cell.2016.03.014 27104977

[73] Maier T Guell M Serrano L . Correlation of mRNA and protein in complex biological samples. FEBS Lett. (2009) 583:3966–74. doi: 10.1016/j.febslet.2009.10.036 19850042

[74] Daeron M Jaeger S Du Pasquier L Vivier E . Immunoreceptor tyrosine-based inhibition motifs: a quest in the past and future. Immunol Rev. (2008) 224:11–43. doi: 10.1111/j.1600-065X.2008.00666.x 18759918

[75] Pasquier B Launay P Kanamaru Y Moura IC Pfirsch S Ruffie C . Identification of FcalphaRI as an inhibitory receptor that controls inflammation: dual role of FcRgamma ITAM. Immunity. (2005) 22:31–42. doi: 10.1016/j.immuni.2004.11.017 15664157

