# Supplementary material for: Identification of a novel, expressed, alternatively spliced FCER1G protein that inhibits receptor function
Source: Front Immunol. 2026 Oct 1;17:1856092. doi: 10.3389/fimmu.2026.1856092 (PMC13668506; doi:10.3389/fimmu.2026.1856092)
Supplement: Supplementary file 1 [file DataSheet2.docx]

**Supplemental Table 1: long-read assemblies analyzed for structural variation in FCER1G**

| Project | Source | N | Project Accession | Cohort Name |
| --- | --- | --- | --- | --- |
| UAB-CASSLE | UAB | 41 | N/A |  |
| CSER | HA | 23 | [DbGAP:phs002307.v1.p1](https://www.ncbi.nlm.nih.gov/projects/gap/cgi-bin/study.cgi?study_id=phs002307.v1.p1) | CSER Project |
| Chinese Pangenome Project | CPG | 57 | [CNCB: PRJCA011422](https://ngdc.cncb.ac.cn/bioproject/browse/PRJCA011422) | Chinese Pangenome Project |
| Arab Pangenome Project | APG | 27 | [SRA:SRP509490](https://trace.ncbi.nlm.nih.gov/Traces/?view=study&acc=SRP509490) | Arab Pangenome Project |
| All of Us | AoU | 917 | N/A | All of Us v8 long-read. |
| Human Pangenome Project | SRA | 63 | [SRA:SRP305758](https://trace.ncbi.nlm.nih.gov/Traces/?view=study&acc=SRP305758) | Human Pangenome Reference Consortium |
| Other SRA PacBio HiFi WGS | SRA | 4 |  | various |

Whole genome long-read data obtained by circular consensus sequencing (CCS) and from the sources listed were used to identify FCER1G structural genomic variants. A number of genomes (≤ 20) from All of Us could not be disambiguated.

**Supplemental Table 2:** Sequences of the probes used to query genomes for copy number variation (CNV) in FCER1G. Probes were based on the hg38 reference genome.

| Marker | Sequence |
| --- | --- |
| FCER1G left flank | ATCTTAGTGTCCTTCACTCGGACTGTAAACCTAAGAATGTTCATTAACCCTCCATTCCTGTTAGATTCAGTCA |
| FCER1G exon 1 | ATGATTCCAGCAGTGGTCTTGCTCTTACTCCTTTTGGTTGAACAAGCAG |
| FCER1G exon 2 | GGAACCAGGAGACTTACGAGACTCTGAAGCATGAGAAACCACCACAGTAG |
| FCER1G right flank | CGTGCCCAGGCTGTAGGAGACTCTGGGTTTGGAGAATCATGGCACCCTCATCCTGTCATTTTCATCAGTTGTGTCTTCA |

**Supplemental Table 3**: Differential expression of FcRγ mRNA isoforms in myeloid cells.

| **A.** RT-qPCR of FcRγ spliceforms  PBMC PMN  Donor Fold Diff Fold Diff  1 404 275  2 886 299  3 471 291  4 1273 417  5 7361 1751  6 571 289  7 1094 277  8 722 205  9 1652 651  10 4611 1262 | | **B.** RNA-seq of FcRγ spliceforms (Bulk cell preps)  Monocytes PMN  Donor Fold Diff Donor Fold Diff  1 140 1 1437  2 218 2 574  3 281 3 338  4 296 4 96  5 268 5 245  6 314 6 1207  7 289 7 296  8 1170 8 107  9 650 9 488  10 362 10 214  11 1485 11 298  12 207 12 118  13 226  14 778  15 153 |
| --- | --- | --- |
| **C.** |  | |

A) Fold difference in WT-FcRγ vs AS-FcRγ mRNA transcripts was determined for PBMCs and PMNs (neutrophils) using RT-qPCR. RT-qPCR was performed using 100 ng of cDNA in a 20 μL reaction volume and the standard 45 cycles in a 7900HT Fast Real-Time PCR System. The Ct values were determined according to manufacturer’s instructions and normalized relative to the internal actin control. The normalized values were used to calculate the fold difference using the 2^-ΔΔCt^ method. B) Fold difference in WT-FcRγ vs AS-FcRγ mRNA transcripts in monocytes and PMNs was determined from the total counts of transcript-specific mRNAs present in bulk RNA-seq datasets (Mistry, 2019; ref 31, and Carlisle, 2021; ref 32). Neutrophil donors without AS-FcRγ mRNA transcripts are excluded from ratiometric analysis. C) Comparison of the ratios of WT-FcRγ to AS-FcRγ mRNA transcripts in monocytes and PMNs from different donors. The ratio of WT-FcRγ to AS-FcRγ mRNA transcript expression varies greatly among donors.

**Supplemental Table 4**. Ratio of WT-FcRγ to AS-FcRγ mRNA in classical and non-classical monocytes.

**A.**	Reads per million

Monocyte subtype	WT-FCER1G	AS-FCER1G	WT/AS Ratio

Classical	718.76	7.93	90.69

Non-Classical	1417.74	19.24	73.70

**B.**	Reads per million

Monocyte subtype	WT-FCER1G	AS-FCER1G	WT/AS Ratio

Classical	1298.06	3.30	392.87

Non-Classical	2337.85	14.12	165.62

Single cell RNA-seq transcript data, clustered as described for Figure 6 and expressed as the number of FcRγ variant-specific mRNAs per million total mRNAs reads. A) Proportion of FcRγ transcripts in the single-cell RNA-seq dataset in Figure 6. B) Proportion of FcRγ transcripts in an independent single-cell RNA-seq dataset (Bashore, 2024; ref 35). Data show that significantly more AS-FcRγ is present in non-classical monocytes compared to classical monocytes (χ^2^ > 100, p < 10^-4^), perhaps related to the higher level of total FCER1G message in non-classical monocytes.
