# Supplementary material for: Identification of a novel, expressed, alternatively spliced FCER1G protein that inhibits receptor function
Source: Front Immunol. 2026 Oct 1;17:1856092. doi: 10.3389/fimmu.2026.1856092 (PMC13668506; doi:10.3389/fimmu.2026.1856092)
Supplement: Supplementary file 2 [file DataSheet1.zip › Supplementary Figure Legends.docx]

**Supplementary Figure Legends:**

**Supplementary Figure 1**. **Single Nucleotide Variants in FcRγ coding regions.**

Whole exome sequencing data from the Regeneron Million Exome and gnomAD v4.1.1 were analyzed as described in Methods. In addition, 76,215 genomes from gnomAD v4 were analyzed to enable assessment of exon5’. Variant call files (VCF) were filtered to retain only those causing non-synonymous coding changes to the cytoplasmic domain exons of WT- and AS-FcRγ. The genomic organization of the *FCER1G* gene is represented on the X-axis. No variant allele frequency (Y-axis) for the canonical *FCER1G* gene greater than 0.15% was identified in any of the populations studied. The common allele is represented in underlined bold.

**Supplementary Figure 2. Expression of AS-FcRγ mRNA in primary cells and cell lines.**

Total RNA was extracted from cells and converted to cDNA using the Superscript preamplification system. cDNA was then used to PCR amplify a 339 base pair cDNA fragment of AS-FcRγ using AS-FcRγ-specific primers and 35 cycles of PCR (see Methods). **A**. Expression of AS-FcRγ mRNA in U937, HL-60, and THP-1 cells but not in the Jurkat T cell line. **B**. Expression of AS-FcRγ in neutrophils (PMN), peripheral blood mononuclear cells (PBMC) and in platelets from a single donor.

**Supplementary Figure 3**. **Validation of the antibody reagents used to determine FcRγ protein expression**. **A**. Map of the peptide sequences used to immunize mice and generate monoclonal antibodies specifically recognizing the canonical WT-FcRγ, the AS-FcRγ variant or a common region in both proteins. **B**. GST-fusion proteins containing the cytoplasmic domains of WT-FcRγ and AS-FcRγ were probed with monoclonal antibodies specific for GST, WT-FcRγ, or AS-FcRγ. **C**. HA-tagged WT-FcRγ or HA-AS-FcRγ proteins were immunoprecipitated from HEK-293 cells transiently expressing the proteins. Precipitates were immunoblotted with mAb 4B1 which is specific for AS-FcRγ and with mAb C20 which recognizes a region common to both proteins. **D**. FcαRI was immunoprecipitated from HEK-293 cells transiently co-expressing FcαRI together with WT-FcRγ or AS-FcRγ. Precipitates were immunoblotted with mAb 7D3 which is specific for WT-FcRγ and with mAb 4B1 which is specific for AS-FcRγ. Immunoprecipitation of FcαRI was also confirmed using anti-FcαRI mAb MIP8a.

**Supplementary Figure 4**. **Variable expression of AS-FcRγ protein in neutrophil (granulocyte) subtypes**. Low density granulocytes (L) and normal density granulocytes (N) were isolated from normal controls and SLE patient peripheral blood samples as described in Methods. Aliquots of cell lysates containing 10μg protein were loaded and separated by SDS-PAGE and immunoblotted for AS-FcRγ using mAb 4B1. Sample lysates in Group 1 were prepared at the NIH and were gifts from Dr Marianna Kaplan. Sample lysates in Group 2 were independently prepared from donors recruited at UAB for data replication.

**Supplementary Figure 5**. **AS-FcRγ stabilizes the surface expression of FcγRIIIa and FcγRIa**. A) FcγRIIIa, the low affinity IgG-binding receptor encoded by *FCGR3A* requires FcRγ for efficient surface expression. HEK-293 cells transiently co-expressing FcγRIIIa and either HA-WT-FcRγ or HA-AS-FcRγ were stained with anti-FcγRIIIa mAb 3G8 and with anti-HA mAb 12CA5 and analyzed by FACS. B) HEK-293 cells transiently co-expressing FcγRIa, the high affinity IgG-binding receptor encoded by *FCGR1A*, and either HA-WT-FcRγ or HA-AS-FcRγ were stained with anti-FcγRIa mAb 32.2 and with anti-HA mAb 12CA5 and analyzed by FACS.

**Supplementary Figure 6**. **AS-FcRγ stabilizes cell surface expression of FcεRI in the FcRγ-deficient RBL-2H3 rat basophilic line**. FcεRIa requires FcRγ for surface expression. The FcRγ-deficient RBL-2H3 variant line was derived from the FcRγ-sufficient RBL-2H3 line by mutagenizing and selecting for cells deficient in FcRγ and FcεRIa expression, as referenced in Methods. FcRγ-deficient cells stably expressing HA-WT-FcRγ or HA-AS-FcRγ were stained with anti-HA mAb 12CA5 or with mIgE and analyzed by FACS.

**Supplementary Figure 7**.  Structural modeling of WT-FcRγ, AS-FcRγ and FcR α-chains.

Structural predictions for the canonical Fc receptor γ-chain (FcRγ) and its alternatively spliced isoform (AS-FcRγ) were generated using the AlphaFold 3 structural prediction server (Google DeepMind) (41). To characterize the structural alterations induced by the alternative splicing event, as well as potential interactions, full-length amino acid sequences for both isoforms were provided as input queries for structural modeling at  [https://alphafoldserver.com/.](https://urldefense.com/v3/__https:/alphafoldserver.com/.__;!!NoSwA-eRAg!ACRwVjV8GVL5I3UIpPeYwrgW6RotzUGxW9pVVj9g0_Eq6Cj2G2L-wkPdGyRS8ZsUajznL2cDwqMzxw22A9JZ4w$)   We modeled WT-FcRγ and AS-FcRγ, alongside the full length amino acid sequence of each of FcαRI, FcγRIIIa, FcγR1a and FcεR1 (in WT-WT, AS-AS and WT-AS combinations). The FcαR1-WT-WT and FcαR1-WT-AS predictions are illustrated.  In contrast to the WT-FcRγ, the membrane-distal cytoplasmic domain of AS-FcRγ appears disordered and is modeled with very low confidence. The predicted template modeling (pTM) and interface predicted template modeling (ipTM) scores were <0.5 in all combinations. AlphaFold3 does not predict structural interaction among the membrane-distal cytoplasmic domains of the α-chains and either WT-FcRγ or AS-FcRγ.

**Supplementary Figure 8. Single cell RNA Seq in Human Neutrophils**

Single cell RNA-seq (scRNA-seq) libraries were obtained from GEO dataset GSE188288 (35). As outlined in Methods, to detect alternatively spliced gamma chain transcripts (AS-FcRγ), a modified gene annotation file re-labeled the AS-FcRγ splice variant of *FCER1G* with a separate gene symbol, which enabled all downstream analysis programs to count AS-FcRγ and all other forms of FcRγ separately. **A**. These datasets were reanalyzed using pipelines for Cell Ranger 9.0.1 (37) and Seurat 5.0.3 (38). Neutrophil subsets were defined as described by Wigerblad (35). **B**. Neutrophils were identified using the expression of *FCGR3B*, *CSFR3R* and *NAMPT* and were classified into NH0, NH1,NH2 and NH3 subsets using the expression of *S100A12*, *S100A6*, *VIM*, *AIF1*, *TXNIP*, *CXCR2*, *MALAT1*, *NEAT1*, *CSF3R*, *IFIT1*, *HERC5* and *ISG15*. U, unclassified subpopulations comprised ~1% of cells. **C**. *FCAR* transcripts shown in light yellow were similarly expressed in all four NH0-NH3 subsets. **D**. The clustering data generated by Seurat were analyzed manually to assess the relative abundance of WT-FcRγ and AS-FcRγ in subpopulations of neutrophils. Interestingly, only 30-31% of *FCAR* single positive cells were *FCER1G* positive consistent with the observation that neutrophils may express FcαR1 without FcRγ-association (47-49).

**Supplementary Figure 9**

**Supplementary Figure 9.** **Sequence comparison of the** **ITAM of FcRγ with** **the CD3ζ ITAM-3 structure**. The FcRγ ITAM, absent in AS FcRγ, has a distribution of serine/threonine residues similar to the CD3ζ ITAM-3 structure which is deleted in the CD3η splice variant of CD3ζ. Phosphorylation of the serine residue at position 51 in the FcRγ ITAM modulates phosphorylation of the tyrosine at position 47 to alter activation (21).
